# Genetic Variants of the DSF Quorum Sensing System in *Stenotrophomonas maltophilia* Influence Virulence and Resistance Phenotypes Among Genotypically Diverse Clinical Isolates

**DOI:** 10.3389/fmicb.2020.01160

**Published:** 2020-06-03

**Authors:** Daniel Yero, Pol Huedo, Oscar Conchillo-Solé, Sònia Martínez-Servat, Uwe Mamat, Xavier Coves, Ferran Llanas, Ignasi Roca, Jordi Vila, Ulrich E. Schaible, Xavier Daura, Isidre Gibert

**Affiliations:** ^1^Institut de Biotecnologia i de Biomedicina (IBB), Universitat Autònoma de Barcelona (UAB), Barcelona, Spain; ^2^Departament de Genètica i de Microbiologia, Universitat Autònoma de Barcelona (UAB), Barcelona, Spain; ^3^Cellular Microbiology, Priority Research Area Infections, Research Center Borstel – Leibniz Lung Center, Borstel, Germany; ^4^Department of Clinical Microbiology-ISGlobal, Hospital Clínic, Universitat de Barcelona, Barcelona, Spain; ^5^Catalan Institution for Research and Advanced Studies (ICREA), Barcelona, Spain

**Keywords:** quorum sensing (QS), diffusible signal factor (DSF), MLST (multilocus sequence typing), *rpf* cluster, biofilm, antibiotic resistance

## Abstract

The pathogenicity of *Stenotrophomonas maltophilia* is regulated in part by its quorum sensing (QS) system. The main QS signaling molecule in *S. maltophilia* is known as diffusible signal factor (DSF), and the *rpf* gene cluster is responsible for its synthesis and perception. Two cluster variants have been previously described, *rpf*-1 and *rpf*-2, which differ basically in the conditions under which DSF is produced. Here, correlations between the *rpf* variant and antibiotic susceptibility, LPS electrophoretic profiles and virulence-related phenotypes were evaluated for a collection of 78 geographically and genetically diverse clinical strains of *S. maltophilia*. In general there were associations between previously established genogroups and the genetic variant of the *rpf* cluster. However, only few genotype-phenotype correlations could be observed. Resistance to the β-lactam antibiotics ceftazidime and ticarcillin was associated with strains carrying the *rpf*-1 variant, whereas strains of variant *rpf*-2, particularly those of genogroup C, showed higher resistance levels to colistin. Strains of variant *rpf*-2 were also significantly more virulent to *Galleria mellonella* larvae than those of *rpf*-1, most likely due to an increased ability of *rpf*-2 strains to form biofilms. A comparative genomic analysis revealed the presence of proteins unique to individual genogroups. In particular, the strains of genogroup C share an operon that encodes for a new virulence determinant in *S. maltophilia* related to the synthesis of an alternative Flp/Tad pilus. Overall, this study establishes a link between the DSF-based QS system and the virulence and resistance phenotypes in this species, and identifies potential high-risk clones circulating in European hospitals.

## Introduction

*Stenotrophomonas maltophilia* are metabolically and genetically diverse Gram-negative bacilli belonging to the γ-proteobacteria class that inhabit a wide range of environmental niches, mainly in association with plant rhizospheres (Ryan et al., 2009; Brooke, 2012). Although *S. maltophilia* are thought to be low-grade pathogens and a rather uncommon cause of bacterial disease in humans, they are increasingly recognized as significant opportunistic pathogens in healthcare settings worldwide, being one of the ten most common organisms found in respiratory tissue samples taken from intensive care unit (ICU) patients in Europe (Zuschneid et al., 2007; Gherardi et al., 2015). Several nosocomial outbreaks of *S. maltophilia* infection and/or colonization have been reported (Denton and Kerr, 1998), and the global prevalence and incidence of these bacteria as human pathogens have increased significantly during the last decade (Chang et al., 2015; Nayyar et al., 2017; Rutter et al., 2017), particularly in cystic fibrosis patients (Hatziagorou et al., 2019). The most common clinical manifestations of *S. maltophilia* infections include pneumonia, bacteremia, wound or urinary-tract infections (Looney et al., 2009; Gales et al., 2019), with crude mortality rates ranging from 14 to 69% in patients with bacteremia, being highest among patients receiving inappropriate initial antimicrobial therapy (Paez and Costa, 2008; Falagas et al., 2009; Kim et al., 2019). Most important, *S. maltophilia* show low susceptibility to many antibiotics, including those commonly used to treat infections by Gram-negative opportunistic pathogens (Sánchez, 2015). Not only have they a variety of intrinsic resistance mechanisms affecting almost all antibiotic classes but they are also able to acquire new resistances via horizontal gene transfer and mutations. The phenomenon of heteroresistance has also been described in *S. maltophilia* as a mechanism to withstand antibiotic treatment (Martínez-Servat et al., 2018). For these reasons, this species has been classified as one of the leading multidrug resistant (MDR) organisms in hospital settings (Brooke, 2014) and has now been included in the global priority list of the top 10 resistant microorganisms (TOTEM) isolated in ICUs (Rello et al., 2019).

The pathogenesis of *S. maltophilia* infections involves several virulence factors, including extracellular enzymes, bacterial motility and biofilm formation (Trifonova and Strateva, 2019). The ability to grow in matrix-enclosed biofilms is an important virulence-related trait of pathogenic bacteria. Among other functions, biofilms facilitate the development of bacterial persistence within the host and increase resistance against the host immune response (González et al., 2018) and antimicrobials (Stewart, 2015), including the last-resort antibiotic colistin (Mulcahy et al., 2008; Kim et al., 2015; Chua et al., 2016). Thus, biofilm formation is regarded as a form of phenotypic resistance (Olivares et al., 2013). *S. maltophilia* forms biofilms on a wide range of biotic and abiotic surfaces, such as indwelling medical devices (Di Bonaventura et al., 2007). Still, very little is known about the genetic mechanisms that control biofilm formation or virulence in these bacteria, and it is not clear whether there is an intrinsic difference in biofilm formation among genomically diverse environmental and clinical isolates (Pompilio et al., 2011). *S. maltophilia* produces a wide range of extracellular enzymes, including lipases, proteases, chitinases, esterases, DNases, and RNases, contributing to its colonization and virulence in different organisms (Ryan et al., 2009; Trifonova and Strateva, 2019). Furthermore, it is well known that *S. maltophilia* synthesizes diverse O-specific polysaccharide structures that constitute immunodominant antigens of the lipopolysaccharide (LPS) molecule and may contribute to the pathogenicity of the bacterium (Waters et al., 2007; Knirel, 2011). A diffusible signal factor (DSF)-mediated quorum sensing (QS) system, encoded by the *rpf* gene cluster, coordinates the regulation of a number of phenotypes in *S. maltophilia*, such as biofilm formation, secretion of proteases, virulence, and tolerance to a range of antibiotics (Fouhy et al., 2007; Ryan et al., 2009; Trifonova and Strateva, 2019). Two genetic variants of the *rpf* gene cluster (*rpf*-1 and *rpf*-2) divide the strains into two phenotypically and genotypically distinct subpopulations (Huedo et al., 2014). Only *rpf*-1 strains produce detectable DSF, which correlates with their ability to control biofilm formation, motility and virulence (Huedo et al., 2014, 2015). However, no clear link between the presence of a specific *rpf* variant and the isolation source of the strains has been found so far (Lira et al., 2017).

An additional important trait of *S. maltophilia* strains is their high genetic diversity (Gherardi et al., 2015) that facilitates adaptation to changing environmental conditions. Numerous genotypic studies of both clinical and environmental strains have revealed considerable variability among *S. maltophilia* isolates (Nicoletti et al., 2011; Pompilio et al., 2011, 2016; Ochoa-Sánchez and Vinuesa, 2017; Patil et al., 2018; Rizek et al., 2018; Steinmann et al., 2018; Bostanghadiri et al., 2019; Mojica et al., 2019). Due to these large genetic divergences, and because previously proposed species are recognized to be closely related to *S. maltophilia*, it is increasingly accepted to present it as the *S. maltophilia* complex (Svensson-Stadler et al., 2012; Patil et al., 2018; Vinuesa et al., 2018). PCR-based multilocus sequence typing (MLST) and whole-genome sequencing (WGS) have clearly shown the existence of several genomic or phylogenetic groups in the *S. maltophilia* complex, and have helped to clarify the taxonomic status of the species (Hauben et al., 1999; Kaiser et al., 2009; Ochoa-Sánchez and Vinuesa, 2017; Steinmann et al., 2018; Gröschel et al., 2020; Mercier-Darty et al., 2020). Even strains isolated from the same patient (Pompilio et al., 2016; Chung et al., 2017) or patients within the same hospital (Valdezate et al., 2004) can belong to distant phylogenetic groups with different phenotypes, probably due to the emergence of adaptive mutations as a result of selective pressure in the clinical environment or inside the host. In addition to interstrain variability, some isolates show cell-to-cell variation within a clonal population as a mechanism to survive environmental fluctuations (Abda et al., 2015). The complex genotypic and phenotypic heterogeneity between and within *S. maltophilia* isolates hampers not only the effectiveness of antimicrobial therapies but also investigations into the virulence and resistance determinants of this species.

To deepen the knowledge and understanding of the resistance and virulence phenotypes displayed by *S. maltophilia*, in a way that is consistent with this high-diversity context, we have studied a panel of genetically diverse clinical *S. maltophilia* isolates with focus on the two genetic variants of the DSF-mediated QS system. Genetic characterization was performed using MLST and *rpf* typing, together with the analysis of shared and unique proteins. Phenotypic analyses included the determination of antibiotic resistance profiles, biofilm formation, protease secretion, cell motility and LPS banding patterns. Moreover, the virulence of the isolates was assessed using two different non-vertebrate model hosts. To our knowledge, a systematic analysis of the population structure based on the two genetic variants of the DSF-mediated QS system and its relationship with the resistance and virulence phenoptypes of clinical *S. maltophilia* strains has not yet been carried out.

## Materials and Methods

### Bacterial Isolates and Growth Conditions

The strain collection used in this study consisted of 78 clinical *S. maltophilia* isolates (Huedo et al., 2014) collected from point prevalence studies in ICUs of geographically distant European hospitals. The strain panel included isolates from sputum, blood and swabs from surgical wound, oropharynx, perineum, vascular ulcer, decubitus ulcer, or a bronchoaspirate (Supplementary Table S1). All clinical strains were isolated from different patients, except for five patients with two isolates each (Supplementary Table S1). Three strains of *S. maltophilia*, namely K279a, D457, and ATCC13637, were included as references. ATCC13637 was purchased from the American Type Culture Collection. Unless otherwise stated, cultures were routinely grown at 37°C in LB broth (10 g/L tryptone, 5 g/L yeast extract and 10 g/L NaCl) on a rotary shaker at 200 rpm. The species of all isolates was confirmed by matrix-assisted laser desorption/ionization time-of-flight (MALDI-TOF) mass spectrometry (data not shown).

### Genome Sequences and Comparative Genomics

Annotated genome sequences of the unique isolates studied here were generated in the study by Gröschel et al. (2020) and are available from NCBI repository under the accession numbers RASX00000000-RAVB00000000. Genome sequences from *S. maltophilia* strains K279a, D457, and ATCC13637 were obtained from the NCBI genome database^1^. The encoded protein sequences were extracted from the genomes and compared to identify core and exclusive proteins. Orthologous protein sequences of the selected *S. maltophilia* strains were assessed by complete reciprocal best hit using UCSC blat (Kent, 2002) as comparison tool and accepting hits with a complete graph as previously reported (Callister et al., 2008) or the largest clique. This algorithm clusters the gene-derived proteins into core (hard and soft) and accessory proteins. Hard core proteins are those found in all genomes, while soft core proteins are found in 95–99% of genomes. Blastp^2^ and the HMM search program jackhmmer (Johnson et al., 2010) were used to identify homologs of the protein sequences using several databases. Prediction of operons was done using the online software and database OperonDB (Pertea et al., 2009).

### Genotypic Characterization and Phylogenetic Analysis

Genotypic characterization was basically performed on the basis of the MLST scheme for *S. maltophilia* (Kaiser et al., 2009). In addition, determination of the *rpf* variant was included in the genotyping as previously described (Huedo et al., 2014). The detailed MLST procedure was obtained from the *S. maltophilia* MLST database^3^. The allele numbers for each locus and the sequence type (ST) were determined online at the *S. maltophilia* MLST database. A combination of the allelic sequences of the seven genes yielded the allelic profile for each isolate. Concatenated sequences of the seven housekeeping genes for each strain were aligned, and a phylogenetic tree was constructed using MEGA version 7.0.21 (Kumar et al., 2016) based on the neighbor-joining algorithm (Tamura–Nei model). Phylogenetic trees were visualized and annotated using the interactive web platform iTOL v3 (Letunic and Bork, 2016). To divide strains into clonal groups according to their MLST allelic profiles and to infer the population structure, BURST analysis (Feil et al., 2004) was used on the basis of five shared loci.

### Analysis of Antimicrobial Susceptibility

The minimum inhibitory concentrations (MICs) of antibiotics were determined using Etest strips following the manufacturer’s instructions (bioMérieux, Madrid, Spain). Briefly, a 0.5-McFarland suspension was used to grow a confluent bacterial lawn on Mueller–Hinton (MH) agar plates (bioMérieux). Seven antibiotics with proven *in vitro* activity against *S. maltophilia* (Falagas et al., 2008; Wang et al., 2014; Chang et al., 2015) were used: ceftazidime, minocycline, levofloxacin, trimethoprim-sulfamethoxazole, amikacin, ticarcillin-clavulanate, and colistin. The MIC values were determined according to the Etest reading guide after 18 h of incubation at 37°C. The susceptibility test results were interpreted according to clinical susceptibility and resistance breakpoints (S) suggested by the Clinical and Laboratory Standards Institute (CLSI) (CLSI, 2019). For *S. maltophilia*, clinical breakpoints are now available for trimethoprim-sulfamethoxazole (*S* ≤ 2 μg/mL), ticarcillin-clavulanate (*S* ≤ 16 μg/mL), ceftazidime (*S* ≤ 8 μg/mL), minocycline (*S* ≤ 4 μg/mL), and levofloxacin (*S* ≤ 2 μg/mL). For amikacin and colistin, CLSI breakpoints for *Pseudomonas aeruginosa* were used with susceptibility cutoffs of 16 and 2 μg/mL, respectively. Isolates with intermediate antibiogram results were considered resistant (non-susceptible). MDR was defined as non-susceptibility to at least one agent in three or more antimicrobial categories (Magiorakos et al., 2012). The MIC50 and MIC90 values represent the MIC value at which ≥50 and ≥90% of the strains within a test population are inhibited, respectively.

In addition to the Etest method, colistin MICs were determined by the broth microdilution (BMD) method in cation-adjusted MH broth (CAMHB) in accordance with CLSI and the European Committee on Antibiotic Susceptibility Testing (EUCAST) joint recommendations (CLSI, 2019; EUCAST, 2019). Briefly, MICs were determined in sterile 96-well plates by two-fold serial dilutions of colistin sulfate (Apollo Scientific Ltd., Batch AS405305) in 100 μL of CAMHB. Bacteria were first grown overnight in CAMHB using CLSI-recommended incubation conditions. After that, 100 μL of the bacterial suspensions with a final optical density at 550 nm (OD_550_) of 0.05 were added to the wells containing the antibiotic dilutions, and the MIC plates were read after 18 h of incubation at 37°C. The MIC was defined as the lowest antibiotic concentration that inhibited 80% of growth (based on OD measurements) in comparison to the control (CLSI, 2015).

### SDS–PAGE and Immunoblotting of Lipopolysaccharides

For identification of identical ladder-like LPS patterns, LPS preparations from proteinase K-digested whole-cell lysates of *S. maltophilia* isolates were separated on 12% sodium dodecyl sulfate polyacrylamide gel electrophoresis (SDS–PAGE) gels and stained with silver nitrate according to the method of Hitchcock and Brown (1983). The lysates were prepared from the biomass of overnight cultures grown on agar plates. For immunoblots, a rabbit polyclonal antibody against heat-inactivated *S. maltophilia* K279a cells was used. In order to detect serological cross-reactivity between O-specific polysaccharides, the antiserum was adsorbed with heat-killed cells of a K279a Δ*rmlBACD* mutant as previously described (Steinmann et al., 2018). The LPS samples were electrotransferred from SDS–PAGE gels onto polyvinylidene difluoride (PVDF) membranes (Millipore, Merck, Darmstadt, Germany), followed by incubation of the blots with adsorbed anti-K279a antibody. The immunoblots were then treated with alkaline phosphatase-conjugated AffiniPure goat anti-rabbit immunoglobulin (Ig)G (H + L) (Dianova, Hamburg, Germany) and developed in the presence of nitroblue tetrazolium and 5-bromo-4-chloro-3-indolylphosphate substrate (Promega).

### Production of Extracellular Protease

Extracellular proteolytic activity was assessed by means of casein hydrolysis on LB agar supplemented with 1% (w/v) skimmed milk. The bacterial suspensions (5 μL each with an OD_550_ of 0.5) were spotted onto the agar, and the plates were incubated at 30°C for 24 h. Proteolytic capacity was evaluated by measuring the diameter of the translucent halos.

### Twitching Motility

Twitching was assessed using subagar stab inoculations (stab assay) from fresh overnight LB agar plates as previously described (Rashid and Kornberg, 2000). To prepare twitching plates, LB medium containing 1% (w/v) Noble agar (Difco^TM^) was used. After incubation at 30°C for 24 h, the agar was removed from the plates, and the twitching zones were visualized by staining with 1% crystal violet (w/v) to measure their diameters. All experiments were conducted in triplicate.

### Biofilm Formation Assays

To determine biofilm formation, overnight cultures of each isolate were grown aerobically (200 rpm) in LB at 30°C, followed by dilution of the cultures into fresh LB to an OD_550_ of 0.1. Sterile untreated 96-well microtiter plates (BrandTech 781662) were inoculated with 200 μL of the bacterial suspensions and incubated at 30°C for 24 h. Prior to biofilm quantification, cell growth was estimated in each well by measuring the optical density at 620 nm (OD_620_) using a microplate reader (Multilabel Plater Reader VICTOR3). Quantification of the amount of biofilm was performed by crystal violet (CV) staining as described previously (Huedo et al., 2014). Briefly, wells containing adhered cells were washed three times with water, fixed at 60°C for 1 h and stained for 15 min with 200 μL of a 0.1% CV solution. The stained biofilms were rinsed with distilled water, allowed to dry at 37°C for 30 min and then extracted with 200 μL of 95% ethanol. The amount of biofilm was quantified by measuring the OD_550_ of dissolved CV using a microplate reader. Biofilm formation (OD_550_ of CV) was normalized to cell growth (OD_620_) and reported as relative biofilm formation. For this quantitative assay, we have used six to eight replicate wells for each isolate. Based on the ratio (OD_550_ of CV)/(OD_620_ of cell growth), the isolates were grouped into three categories to classify their ability to form biofilm: weak (0 to 0.85), moderate (0.85 to 1.5), or strong (>1.5) biofilm formers (Zhuo et al., 2014).

### Virulence in *Caenorhabditis elegans*

Determination of the virulence of *S. maltophilia* strains in the *C. elegans* CF512 infection model was based on the “slow killing” method (Tan et al., 1999). *C. elegans* CF512 [fer-15(b26)II; fem-1(hc17)IV], a strain showing temperature-dependent sterility, was provided by the *Caenorhabditis* Genetics Center (CGC) and routinely maintained on NGM plates (1.7% agar, 50 mM NaCl, 0.25% peptone, 1 mM CaCl_2_, 5 μg/mL cholesterol, 25 mM KH_2_PO_4_, 1 mM MgSO_4_) seeded with *Escherichia coli* OP50 (provided by CGC) at 16°C. Bacterial strains were grown in Brain Heart Infusion (BHI) broth (BD Difco Bacto) overnight at 37°C, and 100 μL of each culture was spread onto a 5.5-cm-diameter NGM agar plate and incubated at 37°C for 24 h. Before usage, plates were cooled down to room temperature, and 15–20 adult hermaphrodite CF512 worms were placed onto plates seeded with the bacterium of interest. Plates were incubated at 25°C (at which worms are sterile) and scored for live worms every 24 h. *E. coli* OP50 was used as a negative control. A worm was considered dead when it no longer responded to touch. Three replicates per strain were prepared.

### Virulence in *Galleria mellonella*

Larvae of *Galleria mellonella* were obtained from our own hatchery, which was established in collaboration with Professor Fernando García del Pino from the Zoology Department at the Universitat Autònoma de Barcelona. To prepare bacterial inocula, *S. maltophilia* isolates were grown overnight at 37°C in 10 mL of BHI in a rotary shaker at 200 rpm. Then, cells were sedimented by centrifugation, washed in PBS and adjusted to contain approximately 5 × 10^5^ CFU in a single dose of 5 μl. Bacterial burden of the doses was confirmed by plating on BHI medium. This inoculum size was selected based on previous studies (Betts et al., 2014; García et al., 2015) that have determined the optimal dose of *S. maltophilia* required to kill *G. mellonella* over a 24–96 h period. Ten larvae per group were infected through the left proleg using a 50-μl Hamilton^®^ Microliter^TM^ syringe with the aforementioned inoculum and incubated in the dark at 30°C in empty petri dishes. Mortality was determined every 24 h. Larvae were considered dead when they no longer responded to touch, which correlates with total melanization (black colored larvae).

### Statistical Analysis

Statistical comparisons between groups were performed using Welch’s *t*-test, i.e., assuming non-equal variance. The associations between two categorical phenotype and genotype variables were investigated using the *z*-test or Fisher’s exact test as appropriate (Fisher’s exact test works better with small sample sizes). The odds ratio (OR) with a corresponding 95% confidence interval (95% CI) for each variable was calculated. In all cases a *p*-value < 0.05 was considered statistically significant, and all tests were two-tailed. Data were analyzed using SPSS 15.0 for Windows (SPSS Inc., Chicago, IL, United States) or GraphPad Prism 5 (GraphPad Software, San Diego, CA, United States). For infection experiments, the time at which 50% of animals were scored as dead (LT50), was calculated using a non-linear regression based on the Hill equation (GraphPad Prism 5).

## Results

### Genetic Diversity of the *S. maltophilia* Strains

**FIGURE 1:**
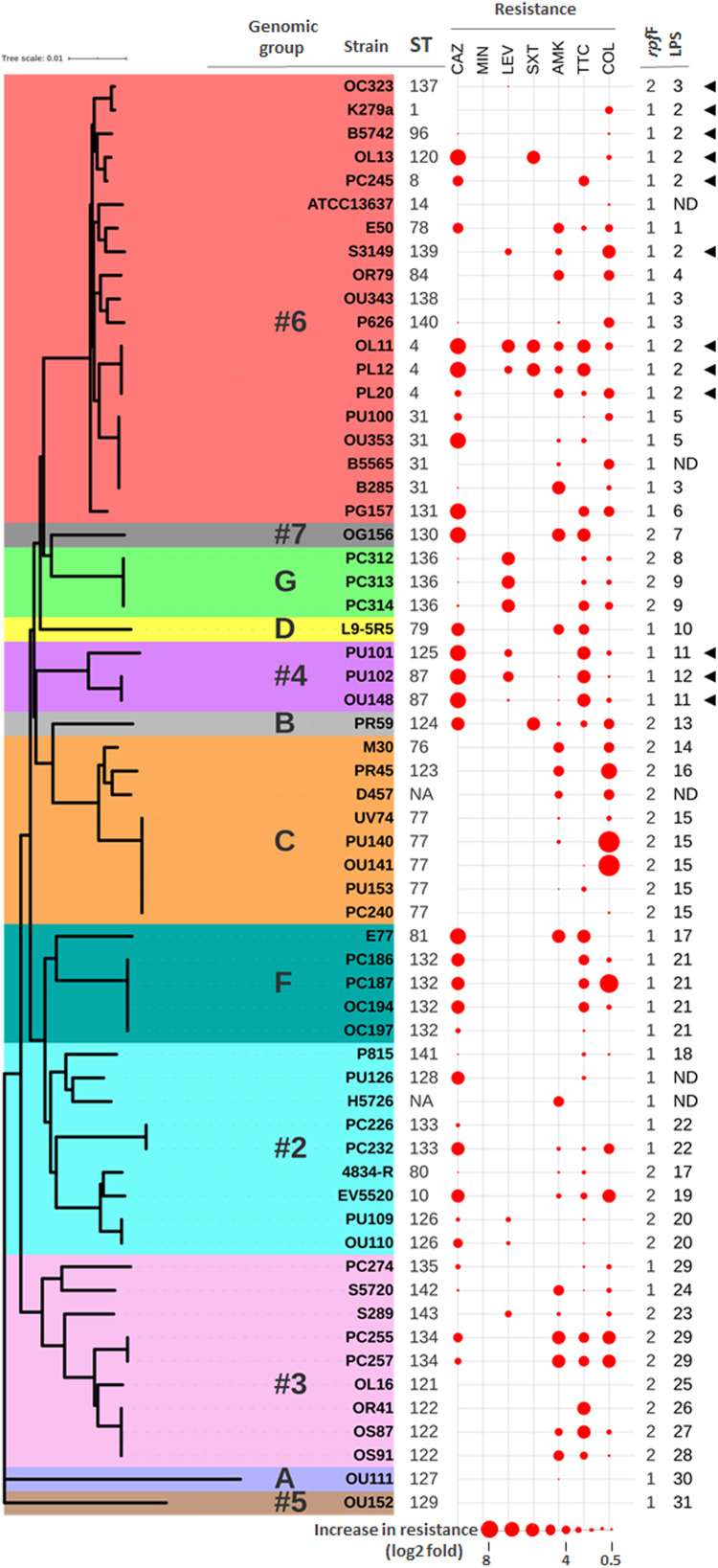
Correlation between genotypes and antibiotic resistance profiles. Neighbor-joining tree based on the concatenated data for all seven MLST loci of the 61 “unique” *S. maltophilia* strains from this study. The ST of each isolate is indicated (NA means not assigned). Previously defined genomic groups and *rpf* variants are also indicated. Data for strain D457 was deduced from its whole genome sequence (NCBI Reference Sequence: NC_017671.1). The antibiotic resistance profiles are displayed as spheres of different sizes in consecutive columns outside the tree. Data are presented as log2 fold increase in resistance with respect to the clinical breakpoint for each antibiotic (abbreviations as in Table 1). Highest values in the dataset have the largest size, and all others are scaled down proportionally. Groups of LPS profiles are also indicated (ND means undetermined). Presence of a K279a-like O-antigen is indicated with black triangles.

To evaluate the genetic diversity and possible relations between the *S. maltophilia* isolates involved in this study, their MLST profiles were first analyzed. The 78 strains, isolated from patients of different European countries, mapped to 37 STs (Figure 1 and Supplementary Table S1). At the date of submission to the *S. maltophilia* MLST database, 30 of these STs were new and designated ST-76 to ST-81 and ST-120 to ST-143. Fifteen isolates (19.2%) belonged to seven previously described STs, whereas 63 (80.8%) belonged to the 30 novel STs, corroborating previous reports on the genotypic heterogeneity of *S. maltophilia*. Although no predominant clonal group was identified by BURST analysis, the three most frequent MLST genotypes were ST-77 (8 isolates), ST-132 (8 isolates), and ST-133 (7 isolates). The remaining STs occurred each with a frequency lower than 5.0%. When the entire *S. maltophilia* MLST database (522 isolates, 367 STs) was analyzed with the BURST algorithm, some of our isolates were part of discrete clonal groups, sharing six (clonal complexes, the most exclusive group definition) to five identical alleles (Supplementary Figure S1). For instance, four strains isolated from three different countries could be grouped into one of the few clonal groups (ST-8) detected so far in the global population of *S. maltophilia* (Supplementary Table S1 and Supplementary Figure S1). Likewise, our predominant ST (ST-77) comprises strains from different European countries and is part of a genetically related group of the clonal complex ST-103 (Supplementary Figure S1). In addition to MLST, *rpf* genotyping (Huedo et al., 2014) revealed that the *rpf*-1 variant was present in a total of 47 strains (60%), while *rpf*-2 was identified in 31 strains (40%) (Supplementary Table S1).

Among the 78 strains studied here, five pairs of isolates were confirmed to be epidemiologically linked. They were either cultured from two sources of the same patient on the same day (four pairs) or originated from samples taken from the same patient at different dates (pair OS91 and OS87) (Supplementary Table S1). In most cases, the paired isolates share the same ST, but isolates PU101 and PU102 are genetically distinct, with only three alleles in common. Also, based on the MLST typing and data from the source of isolation, among the 35 strains collected from Germany, and coming from a single hospital, there seems to be genetically related groups (Supplementary Table S1). In order to avoid interference of clonal strains with correlation tests, those isolates displaying identical MLST type and isolation data (i.e., same source, patient, and date) were considered as the same clone, so that only one representative isolate was selected for further investigations (Supplementary Table S1, highlighted in bold). However, those clonal strains that showed different antibiotic susceptibility profiles (see below) were not excluded from further analyses. Thus, the analysis of correlations between the phenotypic and genotypic parameters studied here was performed on 58 isolates. In addition, we have included the strains K279a, D457, and ATCC13637 as references in all experiments, yielding a total of 61 “unique strains” (36 *rpf*-1 and 25 *rpf*-2).

### Classification of *S. maltophilia* Isolates Into Genomic Groups

For cluster analysis, taxonomic status verification and assignment of the *S. maltophilia* isolates to genomic groups, phylogenetic analyses were carried out based primarily on concatenation of the seven MLST genes (Figure 1 and Supplementary Figure S2). A neighbor-joining tree of the 522 isolates in the MLST database (Supplementary Figure S2) allowed us to cluster all these strains according to the 20 genogroups (#1 to #10 and A to J) as previously established by Hauben et al. (1999), Kaiser et al. (2009), and Mercier-Darty et al. (2020). This phylogenetic analysis showed a lack of clustering by isolation source or geographical origin (Supplementary Figure S2), which stresses again the heterogeneity of *S. maltophilia* isolates, in this case at a worldwide level. Even though most of the strains in the MLST database were of clinical origin, it is noteworthy that almost all environmental strains were clustered in groups #8, #9, and #10. The 78 strains in our panel could be clustered into 12 genogroups (Supplementary Table S1), including the new group G (4 strains) by Mercier-Darty et al. (2020). Therefore, our strain collection provides a good representation of the global diversity of *S. maltophilia*. Nineteen (31%) of the 61 unique isolates were clustered in genogroup #6, including the clonal complex ST-8 (Figure 1). Genomic group #6 comprises the so far largest number of strains in the MLST database (Supplementary Figure S2) and includes the reference strain ATCC13637 and the reference genome strain K279a. The other genogroups with a significant number of isolates in our collection were #2 (19%), #3 (13%) and C (15%), which is in agreement with the frequency of isolates belonging to these groups in the worldwide population. In agreement with previous studies (Corlouer et al., 2017; Mercier-Darty et al., 2020), genogroup #2 represents the most genetically diverse group in our collection. As proposed by Gröschel et al. (2020) and shown in Supplementary Table S1, the strains could be further classified into the monophyletic lineages named Sm1–Sm18 (termed *S. maltophilia sensu lato*) or the more distantly related lineages Sgn1–Sgn4 according to Vinuesa et al. (2018). All our strains, except the phylogenetically distant strain OU111 (genogroup A), belong to the *S. maltophilia sensu lato* cluster. None of them clustered into lineages in which there are isolates mislabeled as *S. maltophilia* or with an unclear taxonomic status, for example the genomic groups #8 and #10 (Ochoa-Sánchez and Vinuesa, 2017). In this respect, our results therefore could provide support for the multispecies concept to explain diversity among isolates of *S. maltophilia*.

Interestingly, there were statistically significant associations between the major MLST-based genomic groups and the *rpf* variants. In the genomic group #6, most of the strains contained the variant *rpf*-1 (*P* < 0.001), whereas the variant *rpf*-2 was predominant in genogroups C (*P* < 0.001) and #3 (*P* < 0.05).

### Identification of Core and Unique Proteins Within *S. maltophilia* Strains and Genogroups

**FIGURE 2:**
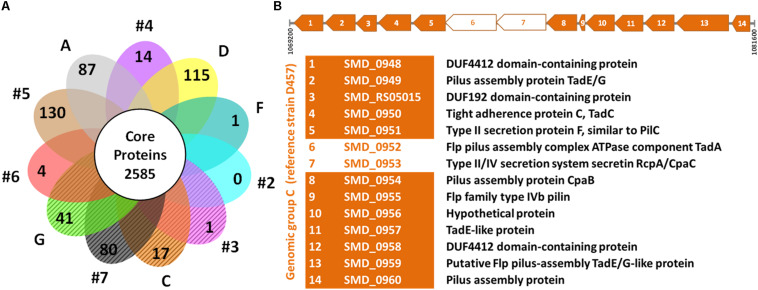
Comparative genomics of *S. maltophilia* genomic groups. **(A)** Flower plot of unique proteins in each genogroup and size of the hard-core proteome. The petals marked with diagonal lines represent the genomic groups where the strains are mainly of the *rpf*-2 variant. The identified unique proteins to each genogroup are listed in Supplementary Table S2. **(B)** Linear map showing the Flp/Tad pilus-encoding gene cluster exclusive to all strains of genogroup C (*rpf*-2), based on strain D457 (NCBI Reference Sequence: NC_017671.1) as the reference genome. The coding sequences (CDS) are numbered from 1 to 14 and the functions of the encoded proteins are shown below the map. Two CDS (empty arrows) were not detected as unique by our algorithm because they have paralogs within each genome.

In addition to the genotypic characterization, the genome-derived protein sequences of 60 unique strains of *S. maltophilia* were compared to identify core and exclusive proteins (assembled genome sequence of PR59 could not be determined). According to our ortholog identification method, the *S. maltophilia* strains included in this comparison share a core genome of 2,585 proteins. If we consider the soft core genome (proteins present in 95–99% of genomes), 349 additional proteins could be added to this set of shared proteins. We also identified the number of exclusive proteins shared by all members of each genogroup (Figure 2A and Supplementary Table S2). The analysis showed that the number of unique proteins to each genogroup varies considerably from genogroup to genogroup. Genogroups #5, #7, D and A clearly stand out having the highest number of unique proteins; this is likely due to these groups being represented by a single strain. It is worth mentioning that all members of genogroup C share 17 unique proteins, 12 of which are part of an operon that encodes a protein translocation system for the assembly of Flp or Tad (tight adherence) pilus (Figure 2B). Moreover, members of genogroup #4 share a unique operon for exopolysaccharide synthesis, and isolates in genogroup G share 41 proteins including peptidases and a transcriptional regulator, in addition to other unique enzymes and several hypothetical proteins (Supplementary Table S2). On the other hand, the comparative analysis did not identify proteins shared exclusively by strains of the *rpf*-1 or *rpf*-2 variants. This result highlights the genetic heterogeneity among clinical *S. maltophilia* isolates and confirms, in addition to their distribution along genotypes, that the origin of the two *rpf* variants in *S. maltophilia* can be explained by horizontal gene transfer.

### Diversity of *S. maltophilia* Isolates Based on Their LPS Profiles

The enormous structural diversity of surface-exposed O-specific polysaccharides of smooth-type LPS is assumed to result from adaptive mechanisms that enable the bacteria to withstand specific conditions in various environmental niches, which in turn provides the possibility of using O-polysaccharides as an epidemiological marker to discriminate between related and unrelated strains within a given species (Rimler, 1990; Aucken and Pitt, 1993; Santamaría et al., 1998). We therefore compared the LPS profiles of our *S. maltophilia* isolates as an additional marker to cluster the strains on the basis of identical LPS patterns in SDS-PAGE, the ladder-like appearance of which is caused by varying numbers of oligosaccharide repeating units in the O-specific polysaccharide (Supplementary Figure S3). As expected, we could observe significant heterogeneity of the LPS banding patterns, whereby none of the isolates investigated here synthesized rough-type LPS lacking the O-specific polysaccharide (Supplementary Figure S3). This was also valid for the strains B5565, H5726 and PU126, but unequivocal evidence for the presence of smooth-type LPS could only be obtained using increasing amounts of LPS in SDS-PAGE (Supplementary Figure S3, *inset*). The three strains were therefore excluded from further analysis of LPS profiles. Based on the electrophoretic mobility of higher molecular mass LPS species, we assigned 76 strains to a total of 31 LPS groups, including 20 isolates with apparently unique LPS patterns (Supplementary Table S1). There were at least two different LPS profiles in each of the genogroups with more than one member, where genogroups F and C with dominating LPS groups 21 and 15, respectively, were the most homogeneous. In summary, apart from some exceptions, we could uncover a good correlation between the LPS profile and the ST of the isolates, which was largely in accord with the phylogenetic tree shown in Figure 2. Furthermore, the LPS samples were screened with a polyclonal antiserum raised against the O-antigen of strain K279a, which has been recently proposed to share similar characteristics with the *S. maltophilia* O8-antigen of a branched tetra-saccharide with three rhamnopyranosyl residues in the main chain and 3-*O*-methylxylose as substituent (Steinmann et al., 2018) (Supplementary Figure S3). Our immunoblot analyses indicated serological cross-reactivity of the antiserum with the O-antigens of all strains of LPS profile group 2 (including *S. maltophilia* K279a), which accounts for 14.5% of all examined isolates. Interestingly, the LPS of all strains of genogroup #4 (3.9%) cross-reacted as well with the antibody, suggesting that these isolates, despite showing different LPS banding patterns, expressed an O-specific polysaccharide either structurally similar or even identical to that of the reference strain K279a. Of note, each of the ten separate groups comprising two to six clonal strains did not only display an identical MLST type, but could also be assigned to one and the same LPS group, which supported the conclusion of epidemiologically linked strains that were isolated at the same hospital in Germany (Supplementary Table S1). All strains that reacted with the O-specific K279a antiserum carried the *rpf*-1 cluster, except for strain OC323 (genetically close to K279a) which encoded the *rpf*-2 variant (Figure 1). This could be in fact another indication that the two *rpf* variants in *S. maltophilia* were acquired by horizontal gene transfer as previously suggested (Huedo et al., 2015), at least for strain OC323 that may have received the *rpf*-2 cluster more recently by recombination events.

### Correlation Between Antibiotic Resistance Phenotypes and Genotypes

The antibiotic resistance pattern of our isolates was evaluated with the β-lactam antibiotics ceftazidime and ticarcillin-clavulanate, and the non-β-lactam antimicrobials amikacin, trimethoprim-sulfamethoxazole, levofloxacin, minocycline and colistin (MIC values in Supplementary Table S1). The MIC values of all antimicrobials were determined by the Etest, with the exception of colistin, which was additionally tested using the BMD method (see section “Materials and Methods”). All strains were susceptible to minocycline, and only 6.5 and 19.7% of the unique strains were non-susceptible to trimethoprim-sulfamethoxazole and levofloxacin, respectively. Conversely, high resistance rates were detected against the antibiotics amikacin (54.1%), ticarcillin-clavulanate (62.3%), and ceftazidime (62.3%), all of them with MIC values reaching >256 μg/mL (Table 1). For colistin susceptibility testing, as expected (Moskowitz et al., 2010), the results of the BMD and Etest methods were significantly different. For our strain collection, the Etest resulted in a colistin MIC50 value of 2 μg/mL corresponding to susceptible strains (susceptibility breakpoint ≤ 2 μg/mL), whereas the BMD method yielded a colistin MIC50 value above the susceptibility breakpoint (>2 μg/mL) (Table 1). Moreover, while the colistin MIC90 value determined by both methods was consistent with a resistant phenotype, it was eightfold lower by the Etest than by the BMD method. This is due to the phenomenon of heteroresistance to colistin displayed by most of the *S. maltophilia* isolates (Martínez-Servat et al., 2018). Finally, taking the recommended BMD method as a reference, 67.3% of the strains could be considered as non-susceptible to colistin.

**TABLE 1:** *In vitro* activity (μg/mL) of seven antimicrobial agents against the 61 “unique” strains of *S. maltophilia* studied here.

**Method**	**Agent**	**Class or sub-class**	**MIC50^a^**	**MIC90^a^**	**Range**	**% of susceptible isolates^b^**
Etest	Ceftazidime (CAZ)	Cephalosporins	16	>256	1–>256	37.7
	Minocycline (MIN)	Tetracyclines	0.25	0.5	0.032–2	100
	Levofloxacin (LEV)	Fluoroquinolones	0.5	16	0.125–>32	80.3
	Trimethoprim-sulfamethoxazole (SXT)	Folate pathway inhibitors	0.25	0.5	0.125–>32	93.5
	Amikacin (AMK)	Aminoglycosides	32	256	2–>256	45.9
	Ticarcillin-clavulanate (TTC)	Penicillins	64	>256	0.5–>256	37.7
	Colistin (COL)	Polymyxins	2	8	0.064–32	75.4
BMD^c^	Colistin	Polymyxins	8	64	<0.25–>256	32.7

*^*a*^MIC90 and MIC50 values were defined as the lowest concentration of the antibiotic on a two-fold scale at which 90 and 50% of the isolates were inhibited, respectively. ^*b*^Susceptibility breakpoints for S. maltophilia established by the Clinical and Laboratory Standards Institute (CLSI, 2019). For amikacin and colistin, CLSI breakpoints for P. aeruginosa were used. Intermediate and resistant strains were grouped together into a non-susceptible category. ^*c*^Broth microdilution.*

**FIGURE 3:**
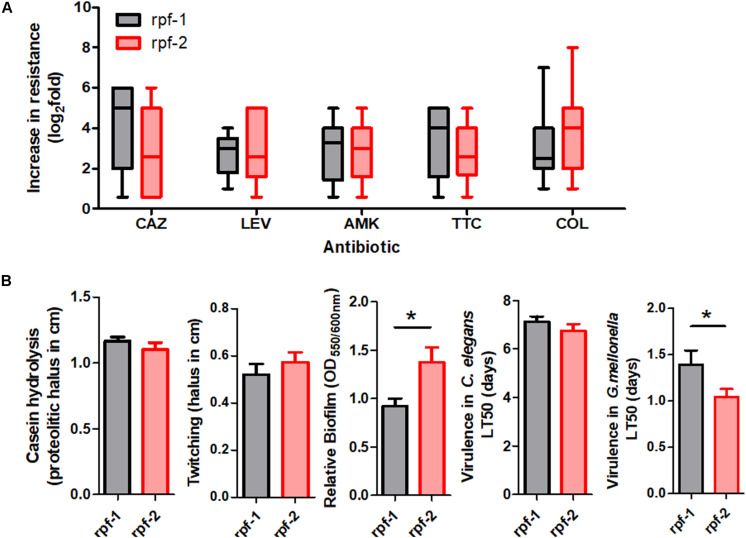
Phenotypic resistance and virulence-related phenotypes within each *rpf*-variant subpopulation of *S. maltophilia*. **(A)** Box and whisker plots (range bars indicate maximum and minimum values) showing log2-fold change increase from susceptibility cut-off for each antibiotic: CAZ, ceftazidime; LEV, levofloxacin; AMK, amikacin; TTC, ticarcillin-clavulanate; COL, colistin. Only resistant isolates were included. **(B)** Profiles for virulence traits of isolates from different *rpf*-variant subpopulations (*p < 0.05; unpaired *t*-test with Welch’s correction).

To further characterize the resistance patterns, the criteria suggested by Magiorakos et al. (2012) for related non-fermenting Gram-negative bacteria were applied. Among the 61 unique isolates, 37 (60.7%) were found to be MDR strains, whereas 24 (39.3%) were not. On the other hand, 22 different antibiotic resistance profiles were observed among the 61 strains (Supplementary Table S1 and Figure 1), the most common being those that combine resistance to ceftazidime, amikacin, ticarcillin-clavulanate, and colistin. Notably, in three out of five strain pairs isolated from the same patient, the members of the same pair showed different resistance profiles (Supplementary Table S1). The two genetically different strains isolated from the oropharinx and the perineum of the same patient, PU101 and PU102, displayed the same resistance profile. These strains were also assigned to different LPS groups. Strain OL11 was the most resistant isolate in our panel (six out of seven antibiotics). It had been isolated with PL12 from different anatomical sites of the same patient. The two strains are genetically identical, possess the same LPS profile (Supplementary Table S1) and are resistant to almost all antibiotics, except that PL12 showed susceptibility to colistin. On the other end, strains OU152, OU343, and OL16, all belonging to different and distant genogroups (Figure 1), were susceptible to all antibiotics tested (Supplementary Table S1). Overall, no significant correlations were observed between the resistance profiles (antibiotic resistance combinations) and the MLST genotypes. However, statistically significant associations could be observed between specific genomic groups and the susceptibility of the isolates to certain antibiotics (Supplementary Table S3). For instance, almost all strains of the genogroup #6 were susceptible to ticarcillin-clavulanate, whereas strains of genogroups #2 and F were mostly susceptible to colistin but ceftazidime resistant. The genetically related strains of genogroups #2 and F showed similar resistance profiles and were therefore considered as a whole in association studies. In addition, we found high-level resistance to levofloxacin in all strains of the new genogroup G. Almost all strains of the genogroup C were susceptible to the β-lactams ceftazidime and ticarcillin-clavulanate, but were quantitatively more resistant to colistin (Supplementary Table S3 and Figure 1). Interestingly, the variant of the *rpf* cluster showed some correlation with the resistance profile of the isolates. Most of the *rpf*-1 strains turned out to be resistant to ceftazidime (*P* = 0.014), and, from a quantitative point of view, the *rpf*-2 resistant strains inclined toward higher MIC values for colistin compared to the *rpf*-1 variant, although without statistical significance (Figure 3A). This analysis also showed higher resistance levels to ceftazidime and ticarcillin-clavulanate among resistant strains of the *rpf*-1 variant.

### Correlation Between Genotypes and Virulence-Related Phenotypes

Virulence-associated phenotypes, including biofilm formation, production of extracellular enzymes and motility, were finally assayed for our strain collection. All isolates were protease and biofilm producers (Supplementary Figure S4). However, the amount of biofilm formed *in vitro* varied considerably among *S. maltophilia* isolates. The *rpf*-2 strain OU110 exhibited the highest biofilm-forming ability. Most of the isolates (44.3%, 27/61) produced moderate biofilms; with a mean of 1.14 and standard deviation of ±0.17 (see “Materials and Methods” section for measurement and classification of biofilms). Strong biofilm formation capacity (mean: 1.95, *SD*: ±0.43) was observed in 24.6% (15/61) of the isolates, whereas the remaining 19 strains (31.1%) showed weak biofilm formation capacity (mean: 0.38, *SD*: ±0.24). With respect to genotypes, we found statistically significant associations (*P* < 0.005) between the isolates of genomic group C and their ability to form biofilm (Supplementary Table S4). On the contrary, 90.9% of strains of genomic groups #6, F and #2 were moderate to weak biofilm producers, though without significant associations. Once again, despite variations in phenotypic traits, the biofilm-forming ability of the strains could be related to the *rpf* variant. Quantitatively, the biofilm formation capacity of *rpf*-2 strains (relative biofilm mean 1.38) was significantly higher (*P* < 0.05) than that of *rpf*-1 strains (relative biofilm mean 0.92) (Figure 3B). With respect to the number of isolates, strains of variant *rpf*-1 are mostly moderate to weak biofilm formers (*P* < 0.001) (Supplementary Table S5). On the other hand, there was no statistically significant correlation of protease secretion and twitching motility with the phylogenetic clustering, except for strains belonging to genogroup C or those that are of the *rpf*-1 variant, which are less casein exoprotease producers (Supplementary Figure S4 and Supplementary Tables S4, S5).

The virulence capacity of the strains was investigated by infecting both the nematode *C. elegans* and larvae of the insect *G. mellonella*. The results were compared and correlated with the assayed virulence-related phenotypes. The *S. maltophilia* strains evaluated in this study were differentially virulent to *C. elegans* in the slow killing assay, with no correlation found with genomic groups (Supplementary Figure S4 and Supplementary Table S4). The strains required 4–10 ten days to kill 50% of the worms (mean LT50 = 6.9 days). Complete killing by *E. coli* OP50, used as a negative control, was achieved after 15 days. No significant correlation was found between the assayed virulence-related phenotypes and the virulence of the strains in the *C. elegans* model. The two least virulent strains in this model, PC274 (LT50 = 10.5 days) and the type strain ATCC13637 (LT50 = 10.0 days), showed low motility and biofilm formation capacity and produced lower amounts of extracellular proteases. Nevertheless, the most virulent strain EV5520 (LT50 = 3.8 days) displayed also weak virulence-related phenotypes, indicating that virulence factors other than those examined here contribute to the infection process in the nematodes. In general the type strain ATCC13637 showed obvious signs of attenuation in all assays (Supplementary Figure S4), as previously reported using a zebrafish infection model (Ferrer-Navarro et al., 2013), most likely due to numerous *in vitro* passages since it was isolated in 1960.

All *S. maltophilia* isolates were virulent to *G. mellonella* larvae in killing assays. The selected inoculum size of 5 × 10^5^ CFU resulted in 20–60% death at 24 h post-inoculation with most strains, while complete killing was observed after 96 h. Some strains were considered to be highly virulent as they were able to kill approximately 60% of the larvae after 24 h of inoculation and reached 100% after 48 h. These highly virulent strains belong to almost all genogroups and *rpf* variants. Nevertheless, on average, *rpf*-2 strains were more virulent than *rpf*-1 strains (*P* = 0.05) on the basis of LT50 values (Figure 3B). The association results showed again no significant correlation between virulence-related phenotypes and the capacity of the strains to kill the larvae.

## Discussion

In recent years *S. maltophilia* has received considerable attention. First, the frequency of MDR phenotypes found among isolates is particularly high, as demonstrated by the panel studied here. Second, *S. maltophilia* is capable of surviving in a wide variety of environmental niches, partly due to its capacity to form biofilms on diverse biotic and abiotic surfaces, including medical devices. Finally, *S. maltophilia* is characterized by a high rate of genomic re-arrangements and hypermutator activity, which allows rapid adaptation to new niches (Berg and Martinez, 2015). With this presentation card, it does not come as a surprise that, despite not being a particularly pathogenic or virulent species, *S. maltophilia* has found its way as an emerging nosocomial pathogen (Brooke, 2012; Adegoke et al., 2017).

Although 60.7% of the clinical strains investigated in this work were MDR, most isolates were susceptible to trimethoprim-sulphamethoxazole and all to minocycline. Monotherapy with trimethoprim-sulfamethoxazole, fluoroquinolones or tetracyclines such as tigecycline, minocycline, and doxycycline remains the most effective antimicrobial treatment against this organism (Chang et al., 2015; Hand et al., 2016). However, resistance to levofloxacin was detected in almost one fifth of the strains evaluated in this study. Even though trimethoprim-sulphamethoxazole is indeed the first choice antibiotic against *S. maltophilia*, resistance to this drug has been observed in 6.5% of the strains included in this work, as it has also been reported in other studies (Valdezate et al., 2001). Minocycline emerges as the best alternative to treat *S. maltophilia*, but more studies are needed to introduce this antibiotic into clinical practice (Hand et al., 2016). On the other hand, colistin is usually the last resort antibiotic for treatment of infections caused by MDR strains, despite its proven toxicity (Petrosillo et al., 2008; Wood et al., 2010). In the present study, in addition to a high proportion of colistin-resistant isolates, we have shown that *S. maltophilia* was capable of developing colistin-resistance heterogeneity, deduced from the observed discrepancies between the two commonly used susceptibility testing methods (Etest vs. BMD) (Lo-Ten-Foe et al., 2007). This is in agreement with our previous, more detailed studies on heteroresistance with four colistin resistant *S. maltophilia* strains (Martínez-Servat et al., 2018). Based on the strain characterization presented here, this phenotypic heterogeneity seems to be widely spread among clinical isolates regardless of their genotypes and resistance profiles. Due to the emergence of resistances to almost all antibiotic classes and the observed heteroresistance phenomenon, combination therapies and novel agents may soon be needed to treat infections caused by MDR *S. maltophilia* strains.

An important finding of this study is that resistance to some antibiotics appears to be influenced by the *rpf* variant. For instance, while the *rpf*-1 subpopulation displayed higher levels of resistance to the β-lactam antibiotics ceftazidime and ticarcillin, strains of the *rpf*-2 group showed higher resistance levels to colistin (Figure 3A). The differential resistance profiles found for the *rpf*-1 and *rpf*-2 subpopulations may be attributable to inherent DSF production and its lack in *rpf*-1 and *rpf*-2 subpopulations, respectively (Huedo et al., 2014). While *rpf*-1 strains synthesize DSF under standard conditions activating the QS, *rpf*-2 strains require detection of exogenous DSF to trigger DSF production (Huedo et al., 2015). In this line, it has been reported that DSF signals stimulate the production and release of outer membrane vesicles (OMVs), which may contain β-lactamases (Devos et al., 2015). Furthermore, some antibiotic resistance mechanisms seem to be controlled by DSF in the plant-growth-promoting model strain *S. maltophilia* R551-3 (*rpf*-1), including the biosynthesis of β-lactamases and multidrug efflux pumps (Alavi et al., 2013). On the other hand, in time-kill curve experiments, *S. maltophilia*’s DSF seemed to influence polymyxin tolerance in *P. aeruginosa*, with addition of DSF leading to an enhanced tolerance to polymyxin B and colistin (Ryan et al., 2008). We have recently shown that naturally occurring DSF signals and synthetic derivatives potentiate the activity of the polymyxin antibiotic colistin against *S. maltophilia* and some members of the *Burkholderia cepacia* complex (Huedo et al., 2019). Accumulation of DSF on the surface may slightly increase the permeabilization of the bacterial membrane due to its lipophilic nature, potentiating colistin activity against *rpf*-1 strains. Worth mentioning, there appear to be associations between MLST genotypes and antibiotic resistance regardless of the *rpf* cluster variant. Strains of genogroup C were mostly susceptible to ticarcillin-clavulanate in addition to ceftazidime. Moreover, all strains of genogroups #4 (*rpf*-1) and G (*rpf*-2) were resistant to levofloxacin, and most strains of genogroups #2 and F (mostly *rpf*-1) were susceptible to amikacin. A significant proportion of β-lactam-resistant isolates arise in *S. maltophilia* through altered expression of β-lactamases (Sánchez, 2015) or amino acid substitutions in efflux pumps (Blanco et al., 2019). Both intrinsic and acquired resistance mechanisms are also involved in resistance to aminoglycosides and quinolones in *S. maltophilia*, such as expression of multidrug−resistant efflux pumps, presence of antibiotic inactivating enzymes or mutations in the genes coding for antibiotic targets (García-León et al., 2014; Sánchez, 2015). The comparative genomic analysis found no unique proteins shared by members of a genomic group that would be involved, in a straightforward, direct way, in antibiotic resistance (Supplementary Table S2). Taken together, these findings confirm that mutations, together with horizontal gene transfer, affect the resistance patterns in *S. maltophilia* isolates beyond genomic diversity.

In addition to antibiotic resistance, the genetic variant of the DSF QS system has an impact on pathogenicity of *S. maltophilia* as well, and many virulence determinants are involved in this process. Biofilm formation is associated with an increase in virulence of *S. maltophilia*. Although the biofilm forming capacity varied considerably among the strains studied here, consistent with previous observations from our group (Huedo et al., 2014), we have shown that strains carrying the *rpf*-2 variant are stronger biofilm producers than strains carrying the *rpf*-1 version (Figure 3B). In a previous study, we showed that, although the protein variant RpfF-2 is functional in DSF synthesis, its permanent repression by RpfC-2 results in the absence of DSF production under the conditions tested. In contrast, the *rpf*-1 subpopulation produces larger amounts of DSF and, in that case, DSF seems to contribute to biofilm dispersion (Huedo et al., 2014). Similar results were observed for the closely related bacterium *Xanthomonas campestris* (encoding a very close RpfF-1 variant), in which DSF also appears to disperse biofilms (Dow et al., 2003). Likewise, in *P. aeruginosa*, a related DSF signal, formerly termed *cis*-DA, displayed a strong dispersion capacity of mature biofilms of a large number of bacterial pathogens (Davies and Marques, 2009). Overall, it seems likely that the absence of DSF production confers on *rpf*-2 members a stronger ability to form biofilms, and this may partially explain why these strains are more virulent in larvae of *G. mellonella*. Pompilio et al. (2016) observed that *S. maltophilia* biofilm formation is directly associated with mortality rate in *G. mellonella*. In general, DSF signaling in *S. maltophilia* regulates factors contributing to virulence, such as motility, biofilm formation and colonization (Alavi et al., 2013; An and Tang, 2018). However, no correlation could be detected when comparing virulence and biofilm formation with casein proteolitic activity or twitching motility, suggesting that the latter attributes are not major virulence traits of *S. maltophilia*, at least in the two infection models tested. On the other hand, no significant association between antibiotic resistance and the amount of biofilm formed could be observed. Thus, our results are similar to those reported by Pompilio et al. (2016) but diverge from those reported by Liaw et al. (2010), who concluded that MDR isolates show higher levels of biofilm formation in comparison to non-MDR strains.

In *S. maltophilia*, the high intra-specific genetic variability, together with a low diversity of the known resistance and virulence factors (Berg and Martinez, 2015) significantly impedes the establishment of relationships with epidemiological relevance. Although we could not uncover significant correlations between all phylogenetic groups and virulence-related phenotypes, we have identified groups of genetically related strains that are scattered throughout European countries and could represent high-risk clones. For instance, although the number of strains tested is small, it can be observed that genogroup C comprises stronger biofilm-forming strains that are almost all resistant to the last-resort antibiotic colistin. However, all these strains are susceptible to other antibiotics tested herein and, thus; there remain good treatment options against infections caused by them. Nevertheless, strains of this group associated with human infections (Supplementary Table S1) have also proven to be highly virulent in different infection models (Ferrer-Navarro et al., 2013; Huedo et al., 2014), and can quickly acquire a resistant phenotype (Alonso and Martínez, 1997; Martínez-Servat et al., 2018). Interestingly, all strains of group C seem to express an additional type of the Flp/Tad pilus encoded by a unique cluster of genes (operon SMD_0948-SMD_0960 in D457). This operon seems to be different from the TadE-like pili gene cluster located at Smlt2867-Smlt2875 in the K279a genome (Crossman et al., 2008), although some components are similar. In strain D457, the closest ortholog to the Flp/Tad pilus in K279a is the system encoded by operon SMD_2505-SMD_2513. The Flp/Tad pilus has been categorized as a subtype of the type II secretion system (Tomich et al., 2007), and the encoding cluster is present in a wide variety of bacterial species, for which it functions as an important virulence factor. It is considered a target of horizontal gene transfer and is essential for biofilm formation (Tomich et al., 2007; Nykyri et al., 2013). To our knowledge, it is the first time that this alternative structure has been found encoded in the genome of strains of *S. maltophilia*, which could contribute to the virulence of these strains. Besides, the genogroup #6 includes the largest subgroup of our strain collection. Of note, the predominance of clinical isolates in genogroup #6 has also been shown by others, mostly in cystic fibrosis patients (Kaiser et al., 2009; Vasileuskaya-Schulz et al., 2011; Corlouer et al., 2017; Ochoa-Sánchez and Vinuesa, 2017; Steinmann et al., 2018). Three of a total of four trimethoprim-sulfamethoxazole resistant isolates of our collection belong to genogroup #6, this antibiotic being a first-line therapeutic choice in *S. maltophilia* infections.

Phylogenomic analyses linked with comparative LPS profiling and O-serotyping have recently revealed correlations between genomic classification of a number of *S. maltophilia* isolates and electrophoretic banding patterns of their LPS in silver-stained polyacrylamide gels and immunoblots with an antibody raised against the O-antigen of the reference strain *S. maltophilia* K279a (Steinmann et al., 2018). The results obtained here confirm the usefulness of LPS profile typing in combination with immunoblotting as a supportive tool for identification of epidemiologically related *S. maltophilia* strains. The LPS-based approach was not only suited to supplement the genotypic characterization of our diverse strain panel, but could also corroborate previous data on the high variability of O-polysaccharide structures among *S. maltophilia* strains.

## Conclusion

Overall, this study shows that the QS system plays a pivotal role in pathogenicity and persistence in *S. maltophilia*. Furthermore, since there are significant correlations between the *rpf* variant and the phylogenetic groups of the isolates based on their MLST profiles (Supplementary Table S4), this genotyping method could be valid as an epidemiological tool. Genome sequencing studies also revealed genes that are exclusive to certain groups of isolates and could contribute to the observed phenotypes, showing that other factors besides DSF synthesis contribute to the virulence and resistance of *S. maltophilia*. More in-depth genomic analysis (e.g., single nucleotide polymorphism detection), together with phenotypic characterization of a larger collection of representative clinical isolates of *S. maltophilia*, will give a much deeper understanding of genetic diversity, phylogeny, population structure and epidemiology of this pathogen. For instance, this will allow the discovery of point mutations involved in antibiotic resistance, for example, in clonal strains isolated from the same patient. Altogether, this could have significant implications in understanding the biology of this species and, even more important, in the management of *S. maltophilia* infections.

## Data Availability Statement

The datasets generated for this study can be found in the https://pubmlst.org/smaltophilia/.

## Author Contributions

DY, PH, SM-S, OC-S, UM, XC, FL, and IR performed the experiments presented in the work. OC-S and DY did the comparative genomics analyses. DY, PH, SM-S, OC-S, and UM participated in the writing of the manuscript. All authors contributed to the design and interpretation of the results, revised the manuscript, and approved it for publication.

## Conflict of Interest

The authors declare that the research was conducted in the absence of any commercial or financial relationships that could be construed as a potential conflict of interest.

## Footnotes

1 https://www.ncbi.nlm.nih.gov/genome/

2 https://blast.ncbi.nlm.nih.gov/Blast.cgi

3 http://pubmlst.org/smaltophilia/

## References

[B1] Abda E. M. Krysciak D. Krohn-Molt I. Mamat U. Schmeisser C. Förstner K. U. (2015). Phenotypic heterogeneity affects *Stenotrophomonas* maltophilia K279a colony morphotypes and β-lactamase expression. *Front. Microbiol.* 6:1373. 10.3389/fmicb.2015.01373 26696982 PMC4667094

[B2] Adegoke A. A. Stenström T. A. Okoh A. I. (2017). Stenotrophomonas maltophilia as an emerging ubiquitous pathogen: looking beyond contemporary antibiotic therapy. *Front. Microbiol.* 8:2276. 10.3389/fmicb.2017.02276 29250041 PMC5714879

[B3] Alavi P. Müller H. Cardinale M. Zachow C. Sánchez M. B. Martínez J. L. (2013). The DSF quorum sensing system controls the positive influence of *Stenotrophomonas maltophilia* on plants. *PLoS One* 8:e67103. 10.1371/journal.pone.0067103 23874407 PMC3715506

[B4] Alonso A. Martínez J. L. (1997). Multiple antibiotic resistance in *Stenotrophomonas maltophilia*. *Antimicrob. Agents Chemother.* 41 1140–1142. 10.1128/AAC.41.5.1140 9145884 PMC163865

[B5] An S. Tang J. (2018). Diffusible signal factor signaling regulates multiple functions in the opportunistic pathogen *Stenotrophomonas maltophilia*. *BMC Res. Notes* 11:569. 10.1186/s13104-018-3690-1 30097057 PMC6086056

[B6] Aucken H. M. Pitt T. L. (1993). Lipopolysaccharide profile typing as a technique for comparative typing of gram-negative bacteria. *J. Clin. Microbiol.* 31 1286–1289. 7684751 10.1128/jcm.31.5.1286-1289.1993 PMC262919

[B7] Berg G. Martinez J. L. (2015). Friends or foes: can we make a distinction between beneficial and harmful strains of the *Stenotrophomonas maltophilia* complex? *Front. Microbiol.* 6:241. 10.3389/fmicb.2015.00241 25873912 PMC4379930

[B8] Betts J. W. Phee L. M. Woodford N. Wareham D. W. (2014). Activity of colistin in combination with tigecycline or rifampicin against multidrug-resistant *Stenotrophomonas maltophilia*. *Eur. J. Clin. Microbiol. Infect. Dis.* 33 1565–1572. 10.1007/s10096-014-2101-3 24781003

[B9] Blanco P. Corona F. Martínez J. L. (2019). Involvement of the RND efflux pump transporter SmeH in the acquisition of resistance to ceftazidime in *Stenotrophomonas maltophilia*. *Sci. Rep.* 9 1–14. 10.1038/s41598-019-41308-9 30894628 PMC6426872

[B10] Bostanghadiri N. Ghalavand Z. Fallah F. Yadegar A. Ardebili A. Tarashi S. (2019). Characterization of phenotypic and genotypic diversity of *Stenotrophomonas maltophilia* strains isolated from selected hospitals in Iran. *Front. Microbiol.* 10:1191. 10.3389/fmicb.2019.01191 31191502 PMC6548850

[B11] Brooke J. S. (2012). *Stenotrophomonas maltophilia*: an emerging global opportunistic pathogen. *Clin. Microbiol. Rev.* 25 2–41. 10.1128/CMR.00019-11 22232370 PMC3255966

[B12] Brooke J. S. (2014). New strategies against *Stenotrophomonas maltophilia*: a serious worldwide intrinsically drug-resistant opportunistic pathogen. *Expert Rev. Anti Infect. Ther.* 12 1–4. 10.1586/14787210.2014.864553 24308713

[B13] Callister S. J. McCue L. A. Turse J. E. Monroe M. E. Auberry K. J. Smith R. D. (2008). Comparative bacterial proteomics: analysis of the core genome concept. *PLoS One* 3:e1542. 10.1371/journal.pone.0001542 18253490 PMC2213561

[B14] Chang Y.-T. Lin C.-Y. Chen Y.-H. Hsueh P.-R. (2015). Update on infections caused by *Stenotrophomonas maltophilia* with particular attention to resistance mechanisms and therapeutic options. *Front. Microbiol.* 6:893. 10.3389/fmicb.2015.00893 26388847 PMC4557615

[B15] Chua S. L. Yam J. K. H. Hao P. Adav S. S. Salido M. M. Liu Y. (2016). Selective labelling and eradication of antibiotic-tolerant bacterial populations in *Pseudomonas aeruginosa* biofilms. *Nat. Commun.* 7:ncomms10750. 10.1038/ncomms10750 26892159 PMC4762895

[B16] Chung H. Lieberman T. D. Vargas S. O. Flett K. B. McAdam A. J. Priebe G. P. (2017). Global and local selection acting on the pathogen *Stenotrophomonas maltophilia* in the human lung. *Nat. Commun.* 8:14078. 10.1038/ncomms14078 28102223 PMC5253648

[B17] CLSI (2015). *Methods for Dilution Antimicrobial Susceptibility Tests for Bacteria that Grow Aerobically. Approved Standard: M07 A10*, 10th Edn. Wayne, PA: Clinical and Laboratory Standards Institute.

[B18] CLSI (2019). *Performance Standards for Antimicrobial Susceptibility Testing: M100-ED29*, 29th Edn. Wayne, PA: Clinical and Laboratory Standards Institute.

[B19] Corlouer C. Lamy B. Desroches M. Ramos-Vivas J. Mehiri-Zghal E. Lemenand O. (2017). *Stenotrophomonas maltophilia* healthcare-associated infections: identification of two main pathogenic genetic backgrounds. *J. Hosp. Infect.* 96 183–188. 10.1016/j.jhin.2017.02.003 28318778

[B20] Crossman L. C. Gould V. C. Dow J. M. Vernikos G. S. Okazaki A. Sebaihia M. (2008). The complete genome, comparative and functional analysis of *Stenotrophomonas maltophilia* reveals an organism heavily shielded by drug resistance determinants. *Genome Biol.* 9:R74. 10.1186/gb-2008-9-4-r74 18419807 PMC2643945

[B21] Davies D. G. Marques C. N. H. (2009). A fatty acid messenger is responsible for inducing dispersion in microbial biofilms. *J. Bacteriol.* 191 1393–1403. 10.1128/JB.01214-08 19074399 PMC2648214

[B22] Denton M. Kerr K. G. (1998). Microbiological and clinical aspects of infection associated with *Stenotrophomonas maltophilia*. *Clin. Microbiol. Rev.* 11 57–80. 9457429 10.1128/cmr.11.1.57 PMC121376

[B23] Devos S. Van Oudenhove L. Stremersch S. Van Putte W. De Rycke R. Van Driessche G. (2015). The effect of imipenem and diffusible signaling factors on the secretion of outer membrane vesicles and associated Ax21 proteins in *Stenotrophomonas maltophilia*. *Front. Microbiol.* 6:298. 10.3389/fmicb.2015.00298 25926824 PMC4396451

[B24] Di Bonaventura G. Stepanović S. Picciani C. Pompilio A. Piccolomini R. (2007). Effect of environmental factors on biofilm formation by clinical *Stenotrophomonas maltophilia* isolates. *Folia Microbiol.* 52 86–90. 10.1007/BF02932144 17571802

[B25] Dow J. M. Crossman L. Findlay K. He Y.-Q. Feng J.-X. Tang J.-L. (2003). Biofilm dispersal in *Xanthomonas campestris* is controlled by cell–cell signaling and is required for full virulence to plants. *Proc. Natl. Acad. Sci. U.S.A.* 100 10995–11000. 10.1073/pnas.1833360100 12960398 PMC196915

[B26] EUCAST (2019). *Breakpoint Tables for Interpretation of MICs and Zone Diameters* Version 9.0. Available online at: http://www.eucast.org/clinical_breakpoints/ (accessed November, 2019).

[B27] Falagas M. E. Kastoris A. C. Vouloumanou E. K. Rafailidis P. I. Kapaskelis A. M. Dimopoulos G. (2009). Attributable mortality of *Stenotrophomonas maltophilia* infections: a systematic review of the literature. *Future Microbiol.* 4 1103–1109. 10.2217/fmb.09.84 19895214

[B28] Falagas M. E. Valkimadi P.-E. Huang Y.-T. Matthaiou D. K. Hsueh P.-R. (2008). Therapeutic options for *Stenotrophomonas maltophilia* infections beyond co-trimoxazole: a systematic review. *J. Antimicrob. Chemother.* 62 889–894. 10.1093/jac/dkn301 18662945

[B29] Feil E. J. Li B. C. Aanensen D. M. Hanage W. P. Spratt B. G. (2004). eBURST: inferring patterns of evolutionary descent among clusters of related bacterial genotypes from multilocus sequence typing data. *J. Bacteriol.* 186 1518–1530. 10.1128/jb.186.5.1518-1530.2004 14973027 PMC344416

[B30] Ferrer-Navarro M. Planell R. Yero D. Mongiardini E. Torrent G. Huedo P. (2013). Abundance of the quorum-sensing factor Ax21 in four strains of *Stenotrophomonas maltophilia* correlates with mortality rate in a New Zebrafish model of infection. *PLoS One* 8:e67207. 10.1371/journal.pone.0067207 23840626 PMC3693955

[B31] Fouhy Y. Scanlon K. Schouest K. Spillane C. Crossman L. Avison M. B. (2007). Diffusible signal factor-dependent cell-cell signaling and virulence in the nosocomial pathogen *Stenotrophomonas maltophilia*. *J. Bacteriol.* 189 4964–4968. 10.1128/JB.00310-07 17468254 PMC1913462

[B32] Gales A. C. Seifert H. Gur D. Castanheira M. Jones R. N. Sader H. S. (2019). Antimicrobial susceptibility of *Acinetobacter calcoaceticus*-*Acinetobacter baumannii* complex and *Stenotrophomonas maltophilia* clinical isolates: results from the SENTRY antimicrobial surveillance program (1997-2016). *Open Forum Infect. Dis.* 6 S34–S46. 10.1093/ofid/ofy293 30895213 PMC6419908

[B33] García C. A. Alcaraz E. S. Franco M. A. Passerini de Rossi B. N. (2015). Iron is a signal for *Stenotrophomonas maltophilia* biofilm formation, oxidative stress response, OMPs expression, and virulence. *Front. Microbiol.* 6:926. 10.3389/fmicb.2015.00926 26388863 PMC4559654

[B34] García-León G. Salgado F. Oliveros J. C. Sánchez M. B. Martínez J. L. (2014). Interplay between intrinsic and acquired resistance to quinolones in *Stenotrophomonas maltophilia*. *Environ. Microbiol.* 16 1282–1296. 10.1111/1462-2920.12408 24447641

[B35] Gherardi G. Creti R. Pompilio A. Di Bonaventura G. (2015). An overview of various typing methods for clinical epidemiology of the emerging pathogen *Stenotrophomonas maltophilia*. *Diagn. Microbiol. Infect. Dis.* 81 219–226. 10.1016/j.diagmicrobio.2014.11.005 25592000

[B36] González J. F. Hahn M. M. Gunn J. S. (2018). Chronic biofilm-based infections: skewing of the immune response. *Pathog. Dis.* 76:fty023. 10.1093/femspd/fty023 29718176 PMC6251518

[B37] Gröschel M. Meehan C. Barilar I. Diricks M. Gonzaga A. Steglich M. (2020). The phylogenetic landscape and nosocomial spread of the multidrug-resistant opportunist *Stenotrophomonas maltophilia*. *Nat. Commun.* 11:2044. 10.1038/s41467-020-15123-0 32341346 PMC7184733

[B38] Hand E. Davis H. Kim T. Duhon B. (2016). Monotherapy with minocycline or trimethoprim/sulfamethoxazole for treatment of *Stenotrophomonas maltophilia* infections. *J. Antimicrob. Chemother.* 71 1071–1075. 10.1093/jac/dkv456 26801080

[B39] Hatziagorou E. Orenti A. Drevinek P. Kashirskaya N. Mei-Zahav M. De Boeck K. (2019). Changing epidemiology of the respiratory bacteriology of patients with cystic fibrosis-data from the European cystic fibrosis society patient registry. *J. Cyst. Fibros.* S1569–1993(19)30838-0. 10.1016/j.jcf.2019.08.006 31492646

[B40] Hauben L. Vauterin L. Moore E. R. Hoste B. Swings J. (1999). Genomic diversity of the genus *Stenotrophomonas*. *Int. J. Syst. Bacteriol.* 49(Pt 4) 1749–1760. 10.1099/00207713-49-4-1749 10555357

[B41] Hitchcock P. J. Brown T. M. (1983). Morphological heterogeneity among *Salmonella* lipopolysaccharide chemotypes in silver-stained polyacrylamide gels. *J. Bacteriol.* 154 269–277. 6187729 10.1128/jb.154.1.269-277.1983 PMC217456

[B42] Huedo P. Kumar V. P. Horgan C. Yero D. Daura X. Gibert I. (2019). Sulfonamide-based diffusible signal factor analogs interfere with quorum sensing in *Stenotrophomonas maltophilia* and *Burkholderia cepacia*. *Future Med. Chem.* 11 1565–1582. 10.4155/fmc-2019-0015 31469336

[B43] Huedo P. Yero D. Martínez-Servat S. Estibariz I. Planell R. Martínez P. (2014). Two different rpf clusters distributed among a population of *Stenotrophomonas maltophilia* clinical strains display differential diffusible signal factor production and virulence regulation. *J. Bacteriol.* 196 2431–2442. 10.1128/JB.01540-14 24769700 PMC4054175

[B44] Huedo P. Yero D. Martinez-Servat S. Ruyra À Roher N. Daura X. (2015). Decoding the genetic and functional diversity of the DSF quorum-sensing system in *Stenotrophomonas maltophilia*. *Front. Microbiol.* 6:761. 10.3389/fmicb.2015.00761 26284046 PMC4517397

[B45] Johnson L. S. Eddy S. R. Portugaly E. (2010). Hidden Markov model speed heuristic and iterative HMM search procedure. *BMC Bioinformatics* 11:431. 10.1186/1471-2105-11-431 20718988 PMC2931519

[B46] Kaiser S. Biehler K. Jonas D. (2009). A *Stenotrophomonas maltophilia* multilocus sequence typing scheme for inferring population structure. *J. Bacteriol.* 191 2934–2943. 10.1128/JB.00892-08 19251858 PMC2681804

[B47] Kent W. J. (2002). BLAT–the BLAST-like alignment tool. *Genome Res.* 12 656–664. 10.1101/gr.229202 11932250 PMC187518

[B48] Kim E. J. Kim Y. C. Ahn J. Y. Jeong S. J. Ku N. S. Choi J. Y. (2019). Risk factors for mortality in patients with *Stenotrophomonas maltophilia* bacteremia and clinical impact of quinolone-resistant strains. *BMC Infect. Dis.* 19:754. 10.1186/s12879-019-4394-4 31462215 PMC6714101

[B49] Kim H. A. Ryu S. Y. Seo I. Suh S.-I. Suh M.-H. Baek W.-K. (2015). Biofilm formation and colistin susceptibility of *Acinetobacter baumannii* isolated from Korean Nosocomial samples. *Microb. Drug Resist. Larchmt. N* 21 452–457. 10.1089/mdr.2014.0236 25714496

[B50] Knirel Y. A. (2011). “Structure of O-Antigens,” in *Bacterial Lipopolysaccharides: Structure, Chemical Synthesis, Biogenesis and Interaction with Host Cells*, eds Knirel Y. Valvano M. (Vienna: Springer), 41–115. 10.1089/mdr.2014.0236

[B51] Kumar S. Stecher G. Tamura K. (2016). MEGA7: molecular evolutionary genetics analysis version 7.0 for bigger datasets. *Mol. Biol. Evol.* 33 1870–1874. 10.1093/molbev/msw054 27004904 PMC8210823

[B52] Letunic I. Bork P. (2016). Interactive tree of life (iTOL) v3: an online tool for the display and annotation of phylogenetic and other trees. *Nucleic Acids Res.* 44 W242–W245. 10.1093/nar/gkw290 27095192 PMC4987883

[B53] Liaw S.-J. Lee Y.-L. Hsueh P.-R. (2010). Multidrug resistance in clinical isolates of *Stenotrophomonas maltophilia*: roles of integrons, efflux pumps, phosphoglucomutase (SpgM), and melanin and biofilm formation. *Int. J. Antimicrob. Agents* 35 126–130. 10.1016/j.ijantimicag.2009.09.015 19926255

[B54] Lira F. Berg G. Martínez J. L. (2017). Double-face meets the bacterial world: the opportunistic pathogen *Stenotrophomonas maltophilia*. *Front. Microbiol.* 8:2190. 10.3389/fmicb.2017.02190 29170656 PMC5684188

[B55] Looney W. J. Narita M. Mühlemann K. (2009). *Stenotrophomonas maltophilia*: an emerging opportunist human pathogen. *Lancet Infect. Dis.* 9 312–323. 10.1016/S1473-3099(09)70083-0 19393961

[B56] Lo-Ten-Foe J. R. Smet A. M. G. A. de Diederen B. M. W. Kluytmans J. A. J. W. van Keulen P. H. J. (2007). Comparative evaluation of the VITEK 2, disk diffusion, Etest, broth microdilution, and Agar dilution susceptibility testing methods for colistin in clinical isolates, including heteroresistant *Enterobacter cloacae* and *Acinetobacter baumannii* strains. *Antimicrob. Agents Chemother.* 51 3726–3730. 10.1128/AAC.01406-06 17646414 PMC2043258

[B57] Magiorakos A.-P. Srinivasan A. Carey R. B. Carmeli Y. Falagas M. E. Giske C. G. (2012). Multidrug-resistant, extensively drug-resistant and pandrug-resistant bacteria: an international expert proposal for interim standard definitions for acquired resistance. *Clin. Microbiol. Infect. Off. Publ. Eur. Soc. Clin. Microbiol. Infect. Dis.* 18 268–281. 10.1111/j.1469-0691.2011.03570.x 21793988

[B58] Martínez-Servat S. Yero D. Huedo P. Marquez R. Molina G. Daura X. (2018). Heterogeneous colistin-resistance phenotypes coexisting in *Stenotrophomonas maltophilia* isolates influence colistin susceptibility testing. *Front. Microbiol.* 9:2871. 10.3389/fmicb.2018.02871 30524420 PMC6262003

[B59] Mercier-Darty M. Royer G. Lamy B. Charron C. Lemenand O. Gomart C. (2020). Comparative whole genome phylogeny of animal, environmental, and human strains confirms the genogroup organization and diversity of the *Stenotrophomonas maltophilia* complex. *Appl. Environ. Microbiol.* 86:e02919-19. 10.1128/AEM.02919-19 32198168 PMC7205487

[B60] Mojica M. F. Rutter J. D. Taracila M. Abriata L. A. Fouts D. E. Papp-Wallace K. M. (2019). Population structure, molecular epidemiology, and β-Lactamase diversity among *Stenotrophomonas maltophilia* isolates in the United States. *mBio* 10:e00405-19. 10.1128/mBio.00405-19 31266860 PMC6606795

[B61] Moskowitz S. M. Garber E. Chen Y. Clock S. A. Tabibi S. Miller A. K. (2010). Colistin susceptibility testing: evaluation of reliability for cystic fibrosis isolates of *Pseudomonas aeruginosa* and *Stenotrophomonas maltophilia*. *J. Antimicrob. Chemother.* 65 1416–1423. 10.1093/jac/dkq131 20430789 PMC2882871

[B62] Mulcahy H. Charron-Mazenod L. Lewenza S. (2008). Extracellular DNA chelates cations and induces antibiotic resistance in *Pseudomonas aeruginosa* biofilms. *PLoS Pathog.* 4:e1000213. 10.1371/journal.ppat.1000213 19023416 PMC2581603

[B63] Nayyar C. Thakur P. Tak V. Saigal K. (2017). *Stenotrophomonas maltophilia*: an emerging pathogen in paediatric population. *J. Clin. Diagn. Res.* 11 DC08–DC11. 10.7860/JCDR/2017/24304.9318 28273966 PMC5324411

[B64] Nicoletti M. Iacobino A. Prosseda G. Fiscarelli E. Zarrilli R. De Carolis E. (2011). *Stenotrophomonas maltophilia* strains from cystic fibrosis patients: genomic variability and molecular characterization of some virulence determinants. *Int. J. Med. Microbiol.* 301 34–43. 10.1016/j.ijmm.2010.07.003 20952251

[B65] Nykyri J. Mattinen L. Niemi O. Adhikari S. Kõiv V. Somervuo P. (2013). Role and regulation of the Flp/Tad pilus in the virulence of pectobacterium atrosepticum SCRI1043 and pectobacterium wasabiae SCC3193. *PLoS One* 8:e73718. 10.1371/journal.pone.0073718 24040039 PMC3767616

[B66] Ochoa-Sánchez L. E. Vinuesa P. (2017). Evolutionary genetic analysis uncovers multiple species with distinct habitat preferences and antibiotic resistance phenotypes in the *Stenotrophomonas maltophilia* complex. *Front. Microbiol.* 8:1548. 10.3389/fmicb.2017.01548 28861062 PMC5562727

[B67] Olivares J. Bernardini A. Garcia-Leon G. Corona F. B. Sanchez M. Martinez J. L. (2013). The intrinsic resistome of bacterial pathogens. *Front. Microbiol.* 4:103. 10.3389/fmicb.2013.00103 23641241 PMC3639378

[B68] Paez J. I. G. Costa S. F. (2008). Risk factors associated with mortality of infections caused by *Stenotrophomonas maltophilia*: a systematic review. *J. Hosp. Infect.* 70 101–108. 10.1016/j.jhin.2008.05.020 18621440

[B69] Patil P. P. Kumar S. Midha S. Gautam V. Patil P. B. (2018). Taxonogenomics reveal multiple novel genomospecies associated with clinical isolates of *Stenotrophomonas maltophilia*. *Microb. Genomics* 4:e000207. 10.1099/mgen.0.000207 30084764 PMC6159553

[B70] Pertea M. Ayanbule K. Smedinghoff M. Salzberg S. L. (2009). OperonDB: a comprehensive database of predicted operons in microbial genomes. *Nucleic Acids Res.* 37 D479–D482. 10.1093/nar/gkn784 18948284 PMC2686487

[B71] Petrosillo N. Ioannidou E. Falagas M. E. (2008). Colistin monotherapy vs. combination therapy: evidence from microbiological, animal and clinical studies. *Clin. Microbiol. Infect.* 14 816–827. 10.1111/j.1469-0691.2008.02061.x 18844682

[B72] Pompilio A. Crocetta V. Ghosh D. Chakrabarti M. Gherardi G. Vitali L. A. (2016). *Stenotrophomonas maltophilia* phenotypic and genotypic diversity during a 10-year colonization in the lungs of a cystic fibrosis patient. *Front. Microbiol.* 7:1551. 10.3389/fmicb.2016.01551 27746770 PMC5044509

[B73] Pompilio A. Pomponio S. Crocetta V. Gherardi G. Verginelli F. Fiscarelli E. (2011). Phenotypic and genotypic characterization of *Stenotrophomonas maltophilia* isolates from patients with cystic fibrosis: genome diversity, biofilm formation, and virulence. *BMC Microbiol.* 11:159. 10.1186/1471-2180-11-159 21729271 PMC3146419

[B74] Rashid M. H. Kornberg A. (2000). Inorganic polyphosphate is needed for swimming, swarming, and twitching motilities of *Pseudomonas aeruginosa*. *Proc. Natl. Acad. Sci. U.S.A.* 97 4885–4890. 10.1073/pnas.060030097 10758151 PMC18327

[B75] Rello J. Kalwaje Eshwara V. Lagunes L. Alves J. Wunderink R. G. Conway-Morris A. (2019). A global priority list of the TOp TEn resistant microorganisms (TOTEM) study at intensive care: a prioritization exercise based on multi-criteria decision analysis. *Eur. J. Clin. Microbiol. Infect. Dis.* 38 319–323. 10.1007/s10096-018-3428-y 30426331

[B76] Rimler R. B. (1990). Comparisons of *Pasteurella multocida* lipopolysaccharides by sodium dodecyl sulfate-polyacrylamide gel electrophoresis to determine relationship between group B and E hemorrhagic septicemia strains and serologically related group a strains. *J. Clin. Microbiol.* 28 654–659. 2332462 10.1128/jcm.28.4.654-659.1990 PMC267771

[B77] Rizek C. F. Jonas D. Garcia Paez J. I. Rosa J. F. Perdigão Neto L. V. Martins R. R. (2018). Multidrug-resistant *Stenotrophomonas maltophilia*: description of new MLST profiles and resistance and virulence genes using whole-genome sequencing. *J. Glob. Antimicrob. Resist.* 15 212–214. 10.1016/j.jgar.2018.07.009 30036694

[B78] Rutter W. C. Burgess D. R. Burgess D. S. (2017). Increasing incidence of multidrug resistance among cystic fibrosis respiratory bacterial isolates. *Microb. Drug Resist. Larchmt. N* 23 51–55. 10.1089/mdr.2016.0048 27326758

[B79] Ryan R. P. Fouhy Y. Garcia B. F. Watt S. A. Niehaus K. Yang L. (2008). Interspecies signalling via the *Stenotrophomonas maltophilia* diffusible signal factor influences biofilm formation and polymyxin tolerance in *Pseudomonas aeruginosa*. *Mol. Microbiol.* 68 75–86. 10.1111/j.1365-2958.2008.06132.x 18312265

[B80] Ryan R. P. Monchy S. Cardinale M. Taghavi S. Crossman L. Avison M. B. (2009). The versatility and adaptation of bacteria from the genus *Stenotrophomonas*. *Nat. Rev. Microbiol.* 7:514. 10.1038/nrmicro2163 19528958

[B81] Sánchez M. B. (2015). Antibiotic resistance in the opportunistic pathogen *Stenotrophomonas maltophilia*. *Front. Microbiol.* 6:658. 10.3389/fmicb.2015.00658 26175724 PMC4485184

[B82] Santamaría M. Gutiérrez-Navarro A. M. Corzo J. (1998). Lipopolysaccharide profiles from nodules as markers of bradyrhizobium strains nodulating wild legumes. *Appl. Environ. Microbiol.* 64 902–906. 16349529 10.1128/aem.64.3.902-906.1998 PMC106344

[B83] Steinmann J. Mamat U. Abda E. M. Kirchhoff L. Streit W. R. Schaible U. E. (2018). Analysis of phylogenetic variation of *Stenotrophomonas maltophilia* reveals human-specific branches. *Front. Microbiol.* 9:806. 10.3389/fmicb.2018.00806 29755435 PMC5932162

[B84] Stewart P. S. (2015). Antimicrobial tolerance in biofilms. *Microbiol. Spectr.* 3. 10.1128/microbiolspec.MB-0010-2014 26185072 PMC4507308

[B85] Svensson-Stadler L. A. Mihaylova S. A. Moore E. R. B. (2012). *Stenotrophomonas* interspecies differentiation and identification by gyrB sequence analysis. *FEMS Microbiol. Lett.* 327 15–24. 10.1111/j.1574-6968.2011.02452.x 22092789

[B86] Tan M. W. Mahajan-Miklos S. Ausubel F. M. (1999). Killing of caenorhabditis elegans by *Pseudomonas aeruginosa* used to model mammalian bacterial pathogenesis. *Proc. Natl. Acad. Sci. U.S.A.* 96 715–720. 10.1073/pnas.96.2.715 9892699 PMC15202

[B87] Tomich M. Planet P. J. Figurski D. H. (2007). The tad locus: postcards from the widespread colonization island. *Nat. Rev. Microbiol.* 5 363–375. 10.1038/nrmicro1636 17435791

[B88] Trifonova A. Strateva T. (2019). *Stenotrophomonas maltophilia* – a low-grade pathogen with numerous virulence factors. *Infect. Dis. Lond. Engl.* 51 168–178. 10.1080/23744235.2018.1531145 30422737

[B89] Valdezate S. Vindel A. Loza E. Baquero F. Cantón R. (2001). Antimicrobial susceptibilities of unique *Stenotrophomonas maltophilia* clinical strains. *Antimicrob. Agents Chemother.* 45 1581–1584. 10.1128/AAC.45.5.1581-1584.2001 11302834 PMC90512

[B90] Valdezate S. Vindel A. Martín-Dávila P. Del Saz B. S. Baquero F. Cantón R. (2004). High genetic diversity among *Stenotrophomonas maltophilia* strains despite their originating at a single hospital. *J. Clin. Microbiol.* 42 693–699. 10.1128/jcm.42.2.693-699.2003 14766838 PMC344440

[B91] Vasileuskaya-Schulz Z. Kaiser S. Maier T. Kostrzewa M. Jonas D. (2011). Delineation of *Stenotrophomonas* spp. by multi-locus sequence analysis and MALDI-TOF mass spectrometry. *Syst. Appl. Microbiol.* 34 35–39. 10.1016/j.syapm.2010.11.011 21247714

[B92] Vinuesa P. Ochoa-Sánchez L. E. Contreras-Moreira B. (2018). GET_PHYLOMARKERS, a software package to select optimal orthologous clusters for phylogenomics and inferring Pan-Genome phylogenies, used for a critical Geno-Taxonomic revision of the genus *Stenotrophomonas*. *Front. Microbiol.* 9:771. 10.3389/fmicb.2018.00771 29765358 PMC5938378

[B93] Wang Y. L. Scipione M. R. Dubrovskaya Y. Papadopoulos J. (2014). Monotherapy with fluoroquinolone or trimethoprim-sulfamethoxazole for treatment of *Stenotrophomonas maltophilia* infections. *Antimicrob. Agents Chemother.* 58 176–182. 10.1128/AAC.01324-13 24145530 PMC3910778

[B94] Waters V. J. Gómez M. I. Soong G. Amin S. Ernst R. K. Prince A. (2007). Immunostimulatory properties of the emerging pathogen *Stenotrophomonas maltophilia*. *Infect. Immun.* 75 1698–1703. 10.1128/IAI.01469-06 17220304 PMC1865680

[B95] Wood G. C. Underwood E. L. Croce M. A. Swanson J. M. Fabian T. C. (2010). Treatment of recurrent *Stenotrophomonas maltophilia* ventilator-associated pneumonia with doxycycline and aerosolized colistin. *Ann. Pharmacother.* 44 1665–1668. 10.1345/apn.1P217 20736426

[B96] Zhuo C. Zhao Q. Xiao S. (2014). The impact of spgM, rpfF, rmlA gene distribution on biofilm formation in *Stenotrophomonas maltophilia*. *PLoS One* 9:e108409. 10.1371/journal.pone.0108409 25285537 PMC4186781

[B97] Zuschneid I. Schwab F. Geffers C. Behnke M. Rüden H. Gastmeier P. (2007). Trends in ventilator-associated pneumonia rates within the German nosocomial infection surveillance system (KISS). *Infect. Control Hosp. Epidemiol.* 28 314–318. 10.1086/507823 17326022

