# Supplementary material for: Genetic Variants of the DSF Quorum Sensing System in Stenotrophomonas maltophilia Influence Virulence and Resistance Phenotypes Among Genotypically Diverse Clinical Isolates
Source: Front Microbiol. 2020 Jun 3;11:1160. doi: 10.3389/fmicb.2020.01160 (PMC7283896; doi:10.3389/fmicb.2020.01160)
Supplement: Supplementary file 1 [file Data_Sheet_1.PDF]

Genetic variants of the DSF quorum sensing system in *Stenotrophomonas maltophilia* influence virulence and resistance phenotypes among genotypically diverse clinical isolates

Daniel Yero, Pol Huedo, Oscar Conchillo-Solé, Sònia Martínez-Servat, Uwe Mamat, Xavier Coves, Ferran Llanas, Ignasi Roca, Jordi Vila, Ulrich E. Schaible, Xavier Daura* and Isidre Gibert*

***Correspondence:** Xavier Daura, Xavier.Daura@uab.cat and Isidre Gibert, Isidre.Gibert@uab.cat

Supplementary tables and figures

Supplementary Tables

Table S1. *S. maltophilia* isolates of this study with their genotypic and phenotypic characteristics.

Strain details[a]				Molecular Typing[b]				LPS group[c]	Antimicrobial resitance profile[d]						
Name	Comments	Clinical Source	origin	ST	GG	Lineage	*Rpf*		CAZ	MIN	LEV	SXT	AMK	TTC	COL
**PU100**		perineum	BE	31	#6	Sm6	1	5	**64**	0.06	0.38	0.25	6	**24**	**16**
**OU353**		oropharinx	BE	31	#6	Sm6	1	5	**>256**	0.06	0.38	0.19	**48**	**64**	1
**B5565**		bronchitis	ES	31	#6	Sm6	1	ND	1	0.25	0.13	0.13	**48**	8	**32**
**B285**		bronchoaspiration	ES	31	#6	Sm6	1	3	**12**	0.09	0.75	0.38	**>256**	6	**8**
**P626**		oropharinx	ES	140	#6	Sm6	1	3	**12**	0.09	0.5	0.19	**32**	12	**32**
**OU343**		oropharinx	BE	138	#6	Sm6	1	3	3	0.09	0.5	0.19	16	3	0.5
**PL12**	Isolated from the same patient	perineum	SL	4	#6	Sm6	1	**2**	**>256**	0.38	**16**	**>32**	**128**	**>256**	0.5
**OL11**		oropharinx	SL	4	#6	Sm6	1	**2**	**>256**	0.38	**>32**	**>32**	**192**	**>256**	**16**
**PL20**		perineum	SL	4	#6	Sm6	1	**2**	**48**	0.19	0.5	0.38	**192**	**64**	**32**
**ATCC13637**	Reference strain	oropharinx	EEUU	14	#6	Sm6	1	ND	0.75	0.13	0.38	0.19	2	0.5	**4**
**S3149**		surgical wound	ES	139	#6	Sm6	1	**2**	4	0.25	**12**	0.25	**96**	6	**64**
**E50**		sputum	ES	78	#6	Sm6	1	1	**128**	0.13	0.5	0.19	**256**	**64**	**16**
**B5742**		bronchoaspiration	ES	96	#6	Sm6	1	**2**	**12**	0.06	0.38	0.5	4	8	**4**
**PC245**	Considered the same clone	perineum	DE	8	#6	Sm6	1	**2**	**128**	0.13	0.5	0.25	16	**256**	2
PC246		perineum	DE	8	#6	Sm6	1	**2**	**256**	0.09	0.5	0.25	12	**>256**	2
**OL13**		oropharinx	SL	120	#6	Sm6	1	**2**	**>256**	0.75	0.75	**>32**	8	16	**8**
**OR79**		oropharinx	FR	84	#6	Sm6	1	4	8	0.09	0.5	0.25	**256**	12	**32**
**K279a**	Reference strain	blood infection	GB	1	#6	Sm6	1	**2**	3	0.75	1.5	0.38	16	4	**16**
OC322	Considered the same clone	oropharinx	DE	137	#6	Sm6	2	**2**	1.5	0.38	2	0.25	2	4	1
**OC323**		oropharinx	DE	137	#6	Sm6	2	**2**	2	0.25	**3**	0.25	3	4	0.5
**PG157**		perineum	SL	131	#6	Sm6	1	6	**>256**	0.09	0.38	0.75	8	**256**	**32**
**OG156**		oropharinx	SL	130	#7	Sm18	2	7	**>256**	0.13	0.38	0.25	**>256**	**>256**	2
**PC312**		perineum	DE	136	G	Sm17	2	8	**12**	0.50	**>32**	0.25	16	**64**	**8**
**PC314**	Considered the same clone	perineum	DE	136	G	Sm17	2	9	**16**	1.50	**>32**	0.38	8	**256**	**16**
PC315		perineum	DE	136	G	Sm17	2	9	**16**	1.50	**>32**	0.38	8	**48**	**16**
**PC313**		perineum	DE	136	G	Sm17	2	9	**12**	0.75	**>32**	0.38	8	**64**	**8**

**L9-5R5**		clinical	ES	79	D	Sm12	1	10	**256**	0.13	0.19	0.5	**256**	**256**	<0.25
**OU148**		oropharinx	BE	87	#4	Sm7	1	**11**	**>256**	0.50	**4**	0.19	**24**	**>256**	**8**
**PU102**	Isolated from the same patient	oropharinx	BE	87	#4	Sm7	1	**12**	**>256**	0.19	**32**	0.5	**24**	**>256**	**4**
**PU101**		perineum	BE	125	#4	Sm7	1	**11**	**>256**	0.25	**16**	0.38	16	**>256**	**8**
OC195	Considered the same clone	oropharinx	DE	132	F	Sm10	1	21	**24**	0.09	0.38	0.19	6	**32**	1
OC196		oropharinx	DE	132	F	Sm10	1	21	**32**	0.09	0.38	0.13	8	**32**	1
**OC197**		oropharinx	DE	132	F	Sm10	1	21	**32**	0.09	0.38	0.13	4	**32**	1
**OC194**		oropharinx	DE	132	F	Sm10	1	21	**256**	0.19	0.38	0.19	4	**256**	**8**
**PC187**		perineum	DE	132	F	Sm10	1	21	**256**	0.19	0.38	0.19	4	**256**	**256**
PC184	Considered the same clone	perineum	DE	132	F	Sm10	1	21	**256**	0.09	0.25	0.25	8	**64**	1
PC185		perineum	DE	132	F	Sm10	1	21	**256**	0.19	0.38	0.19	6	**256**	**4**
**PC186**		perineum	DE	132	F	Sm10	1	21	**256**	0.19	0.25	0.19	6	**256**	**8**
**E77**		sputum	ES	81	F	Sm17	1	17	**>256**	0.09	0.19	0.19	**>256**	**>256**	<0.25
**PU126**		perineum	BE	128	#2	Sm9	1	ND	**256**	0.19	0.38	0.19	3	**48**	2
**H5726**		hematologic neoplasia	ES	NA	#2	Sm9	1	ND	1.5	0.13	0.25	0.13	**256**	3	<0.25
**P815**		oropharinx	ES	141	#2	Sm9	1	18	**12**	0.19	0.25	0.19	12	**48**	**4**
**PU109**	Isolated from the same patient	perineum	BE	126	#2	Sm15	2	20	**24**	0.75	**8**	0.25	4	**32**	1
**OU110**		oropharinx	BE	126	#2	Sm15	2	20	**96**	1.00	**6**	0.19	4	**24**	0.5
**EV5520**		hospital environmental	ES	10	#2	Sm15	2	19	**256**	0.19	0.25	0.13	**64**	**96**	**64**
**4834-R**		clinical	ES	80	#2	Sm15	2	17	**12**	0.13	0.38	0.19	**32**	**48**	1
**PC226**		perineum	DE	133	#2	Sm8	1	22	**24**	0.19	0.75	0.19	8	8	1
PC227	Considered the same clone	perineum	DE	133	#2	Sm8	1	22	**24**	0.19	0.75	0.13	16	12	**32**
PC228		perineum	DE	133	#2	Sm8	1	22	**24**	0.19	0.5	0.13	16	**24**	**8**
PC229		perineum	DE	133	#2	Sm8	1	22	**24**	0.19	0.5	0.13	6	**24**	**8**
PC230		perineum	DE	133	#2	Sm8	1	22	**24**	0.09	0.5	0.13	8	16	**8**
PC231		perineum	DE	133	#2	Sm8	1	22	**24**	0.19	0.5	0.13	**24**	16	**8**
**PC232**		perineum	DE	133	#2	Sm8	1	22	**256**	0.13	1.00	0.13	**48**	**48**	**32**
**PR59**		perineum	FR	124	B	ND	2	13	**256**	0.09	0.19	**>32**	**48**	**96**	**32**
**PR45**		perineum	FR	123	C	Sm4a	2	16	2	0.09	0.5	0.19	**256**	6	**128**
**D457**	Reference strain	bronchoaspiration	ES	NA	C	Sm4a	2	ND	1.5	0.19	0.75	0.13	**128**	4	**32**
**M30**		decubitus ulcer	ES	76	C	Sm4a	2	14	1.5	0.03	0.19	0.13	**256**	12	**32**
PC237	Considered the same clone	perineum	DE	77	C	Sm4a	2	15	1.5	0.09	1.5	0.19	8	3	**8**
PC238		perineum	DE	77	C	Sm4a	2	15	0.75	0.09	2	0.25	8	4	**8**

PC239		perineum	DE	77	C	Sm4a	2	15	0.75	0.19	2	0.19	8	4	**4**
**PC240**		perineum	DE	77	C	Sm4a	2	15	1	0.25	2	0.25	8	2	**4**
**PU153**		perineum	BE	77	C	Sm4a	2	15	3	0.13	1.5	0.38	**24**	**64**	0.5
**OU141**	Isolated from the same patient	oropharinx	BE	77	C	Sm4a	2	15	0.75	0.19	0.75	0.25	16	**24**	**>256**
**PU140**		perineum	BE	77	C	Sm4a	2	15	0.75	0.13	1	0.25	**48**	2	**>256**
**UV74**		vascular ulcera	ES	77	C	Sm4a	2	15	1.5	0.38	1.5	0.13	**32**	3	**8**
**S289**		sputum	ES	143	#3	Sm3	2	23	1.5	0.50	**12**	0.38	**48**	8	**8**
**S5720**		sputum	ES	142	#3	Sm5	1	24	**16**	0.09	0.38	0.25	**256**	**24**	**8**
PC271	Considered the same clone	perineum	DE	135	#3	Sm5	1	29	**24**	0.05	0.5	0.13	3	**32**	**4**
PC272		perineum	DE	135	#3	Sm5	1	29	**32**	0.05	0.5	0.13	4	**24**	**16**
PC273		perineum	DE	135	#3	Sm5	1	29	**48**	0.05	0.75	0.19	3	**32**	**16**
**PC274**		perineum	DE	135	#3	Sm5	1	29	**32**	0.05	0.5	0.19	6	**32**	**8**
**OS91**	Isolated from the same patient	oropharinx	FR	122	#3	Sm3	2	28	3	0.09	0.5	0.13	**256**	**128**	**4**
**OS87**		oropharinx	FR	122	#3	Sm3	2	27	3	0.09	0.38	0.19	**128**	**>256**	**8**
**OR41**		oropharinx	FR	122	#3	Sm3	2	26	2	0.09	0.5	0.19	16	**>256**	2
**OL16**		oropharinx	SL	121	#3	Sm3	2	25	4	0.25	2	0.38	8	16	1
PC256	Considered the same clone	perineum	DE	134	#3	Sm3	2	29	**64**	0.19	0.75	0.19	**>256**	**>256**	**64**
**PC257**		perineum	DE	134	#3	Sm3	2	29	**48**	0.19	0.75	0.19	**>256**	**256**	**64**
PC254	Considered the same clone	perineum	DE	134	#3	Sm3	2	29	**48**	0.38	1.5	0.25	**>256**	**>256**	**64**
**PC255**		perineum	DE	134	#3	Sm3	2	29	**96**	0.19	1	0.19	**>256**	**256**	**64**
**OU152**		oropharinx	BE	129	#5	Sm11	1	30	0.75	0.06	0.13	0.19	2	8	0.25
**OU111**		oropharinx	BE	127	A	Sgn4	1	31	6	0.06	0.25	0.38	**24**	16	0.25

[a] Isolates considered as "unique strains", used to explore associations between genotypes and resistance phenotypes, are indicated in bold. Confirmed epidemiologically related strains, isolated from the same patient, are indicated in bold. Origin: BE (Belgium), ES (Spain), SL (Slovenia), FR (France), EEUU (United States), Germany (DE), United Kingdom (GB). For more details see reference (Huedo et al., 2014).

[b] Sequence type (ST) determined based on the analysis of the sequences of the seven housekeeping genes *atpD, gapA, guaA, mutA, nuoD, ppsA* and *recA* of the MLST scheme for *S. maltophilia.* NA: not assigned. Genomic group (GG) assigned according to Kaiser *et al*. (2009), Hauben *et al*. (1999) and Mercier-Darty *et al*. (2020). Lineages according to Gröschel *et al*. (2020). The *rpf* variants 1 and 2 were determined by sequence alignment against reference strains (Huedo et al., 2014). ND: no determined.

[c] Groups of LPS profiles. The presence of a K279a-like O-antigen is indicated in bold. ND: undetermined.

[d] MIC (µg/mL) using the Etest method, except for colistin, for which this value was determined by the broth microdilution method. Antibiotics: CAZ (Ceftazidime), MIN (Minocycline), LEV (Levofloxacin), SXT (Trimethoprim-sulfamethoxazole), AMK (Amikacin), TTC (Ticarcillin-clavulanate), COL (Colistin). MIC values indicating non-susceptibility to the corresponding antibiotic are shown in bold.

Table S2. Unique proteins shared by all members of each genogroup. Only genomic groups with more than one isolate were analyzed. Predicted operons are indicated in bold.

Genomic Grup	Reference strain	Locus Tag	Annotation and predicted function
#6	K279a	Smlt0249	hypothetical protein
		Smlt1357	conserved exported hypothetical protein
		Smlt2598	wall associated protein, similar to type IV secretion protein Rhs in *Xanthomonas arboricola pv. juglandis*
		Smlt4146	conserved exported hypothetical protein
C	D457	**SMD_0948**	**DUF4412 domain-containing protein**
		**SMD_0949**	**pilus assembly protein TadE/G**
		**SMD_RS05015**	**DUF192 domain-containing protein**
		**SMD_0950**	**Tight adherence protein C, TadC**
		**SMD_0951**	**type II secretion protein F, similar to pilus assembly protein PilC**
		**SMD_0954**	**pilus assembly protein CpaB**
		**SMD_0955**	**Flp family type IVb pilin**
		**SMD_0956**	**hypothetical protein**
		**SMD_0957**	**TadE-like protein**
		**SMD_0958**	**DUF4412 domain-containing protein, similar to apolipoprotein D and lipocalin family protein**
		**SMD_0959**	**putative Flp pilus-assembly TadE/G-like protein**
		**SMD_0960**	**pilus assembly protein**
		SMD_RS05080	hypothetical protein
		SMD_1784	GCN5 family acetyltransferase
		SMD_2150	superfamily I DNA/RNA helicase protein
		SMD_RS20725	EcsC family protein (ABC transport system)
		SMD_4190	DUF2628 domain-containing protein
#4	OU148	D7Y19_02070	hypothetical protein
		D7Y19_02080	hypothetical protein
		D7Y19_04170	hypothetical protein
		D7Y19_05530	similar to Phophatidylinositol-4-phosphate 5-kinase in *Alcanivorax dieselolei*
		D7Y19_06245	hypothetical protein
		D7Y19_09250	class I SAM-dependent methyltransferase
		**D7Y19_15075**	**glycosyltransferase family 1 protein**
		**D7Y19_15080**	**similar to oligosaccharide repeat unit polymerase in *Citrobacter freundii***
		**D7Y19_15085**	**acyltransferase**
		**D7Y19_15090**	**glycosyltransferase**
		**D7Y19_15095**	**glycosyltransferase**
		**D7Y19_15100**	**flippase**
		D7Y19_17785	hypothetical protein
		D7Y19_19245	hypothetical protein
#3	OL16	D7U77_16075	hypothetical protein
F	E77	D7Y58_17670	DUF4288 domain-containing protein

G	PC312	D7Y44_00250	hypothetical protein
		D7Y44_00270	hypothetical protein
		D7Y44_02265	hypothetical protein
		D7Y44_04225	hypothetical protein
		**D7Y44_06200**	**hypothetical protein**
		**D7Y44_06205**	**hypothetical protein**
		D7Y44_08480	hypothetical protein
		D7Y44_08630	NAD-dependent DNA ligase
		D7Y44_09125	hypothetical protein
		D7Y44_09240	hypothetical protein
		D7Y44_09455	HAD family hydrolase
		**D7Y44_09470**	**hypothetical protein**
		**D7Y44_09475**	**hypothetical protein**
		**D7Y44_10630**	**hypothetical protein**
		**D7Y44_10635**	**hypothetical protein**
		**D7Y44_10645**	**hypothetical protein**
		D7Y44_10670	hypothetical protein
		D7Y44_10750	N-acetyltransferase
		D7Y44_10980	hypothetical protein
		**D7Y44_11715**	**hypothetical protein**
		**D7Y44_11720**	**CPBP family intramembrane metalloprotease**
		**D7Y44_12610**	**hypothetical protein**
		**D7Y44_12615**	**ImmA/IrrE family metallo-endopeptidase**
		**D7Y44_12620**	**hypothetical protein**
		D7Y44_12660	hypothetical protein
		D7Y44_12895	colicin immunity protein
		D7Y44_13150	hypothetical protein
		D7Y44_16200	hypothetical protein
		**D7Y44_16640**	**DMT family transporter**
		**D7Y44_16645**	**XRE family transcriptional regulator**
		D7Y44_18100	DUF2806 domain-containing protein
		D7Y44_18470	hypothetical protein
		**D7Y44_18740**	**hypothetical protein**
		**D7Y44_18745**	**AAA family ATPase**
		**D7Y44_18755**	**hypothetical protein**
		**D7Y44_18765**	**hypothetical protein**
		**D7Y44_18770**	**DUF1508 domain-containing protein**
		D7Y44_19315	hypothetical protein
		D7Y44_20295	FAD-binding oxidoreductase
		D7Y44_20685	hypothetical protein
		D7Y44_21535	hypothetical protein

Table S3. Associations of main genomic groups and *rpf* typing with antibiotic resistance.

Genomic groups	Number of isolates[b]													
	CAZ		LEV		SXT		AMK		TTC		COL		MDR	
	S	R	S	R	S	R	S	R	S	R	S	R	No	Yes
#6	7	12	15	4	16	3	9	10	11	8	5	14	8	11
Association[a]	ns		ns		ns		ns		3.438 (1.1-10.65) *P*=0.0452		ns		ns	
#2 + F	1	13	12	2	14	0	9	5	2	12	8	6	4	10
Association[a]	0.087 (0.01-0.72) *P*=0.01		ns		ns		ns		ns		3.89 (1.12-13.5) *P*=0.048		ns	
C	8	0	8	0	8	0	2	6	6	2	1	7	8	0
Association[a]	42.23 (2.29-777.6) *P*=0.0002		ns		ns		ns		6.353 (1.16-34.83) *P*=0.044		ns		38.64 (2.1-710.0) *P*=0.0002	
#3	5	4	8	1	9	0	3	6	2	7	2	7	2	7
Association[a]	ns		ns		ns		ns		ns		ns		ns	
Other groups	2	9	6	5	10	1	6	5	2	9	4	7	4	9
Total	23	38	49	12	57	4	29	32	23	38	20	41	24	37
rpf types	Number of isolates[b]													
rpf-1	9	27	30	6	33	3	17	19	14	22	12	24	13	23
rpf-2	14	11	19	6	24	1	12	13	9	16	8	17	11	14
Association[a]	0.262 (0.09-0.78) *P*=0.014		ns		ns		ns		ns		ns		ns	
Total	23	38	49	12	57	4	29	32	23	38	20	41	24	37

[a]Odds ratio (CI) *P* value. The odds ratio with the corresponding 95% confidence interval (CI) for each variable was calculated by univariate analysis using Fisher's exact test. For zero values, the odds ratio was calculated by adding 0.5 to each value. ns: no significant association. For associations with *rpf* typing, the odds ratio corresponds to the *rpf*-1 variant.

[b]Number of resistant (R) or susceptible (S) isoltes to antibiotics CAZ (Ceftazidime), LEV (Levofloxacin), SXT (Trimethoprim-sulfamethoxazole), AMK (Amikacin), TTC (Ticarcillin-clavulanate), COL (Colistin). MDR: multidrug resistant based on tested antibiotic classes.

Table S4. Associations of main genomic groups with *rpf* typing and virulence-related phenotypes.

Genomic groups	Number of isolates											
	rpf variant		Exoprotease production[b]		Twitching motility[b]		Biofilm		Virulence in *C. elegans*[c]		Virulence in *G. mellonella*[c]	
	1	2	High	Low	High	Low	Strong	Moderate-weak	Weak (LT50>7)	Moderate (LT50<7)	Moderate (LT50>1.2)	Strong (LT50<1.2)
#6	18	1	10	9	15	4	2	17	8	11	5	13
Association[a]	19.8 (2.4-162.2) *P*=0.0004		ns		ns		ns		ns		ns	
#2 + F	10	4	10	4	10	4	1	13	9	5	10	4
Association[a]	ns		ns		ns		ns		ns		ns	
C	0	8	1	7	8	0	6	2	5	3	2	6
Association[a]	0.023 (0.001-0.43) P=0.0002		0.067 (0.007-0.59) *P*=0.046		ns		12.0 (2.12-67.8) *P*=0.0035		ns		ns	
#3	2	7	6	3	6	3	4	5	5	4	4	5
Association[a]	0.13 (0.02-0.68) *P*=0.02		ns		ns		ns		ns		ns	
Others	6	5	10	1	6	5	2	9	4	7	8	3
Total	38	23	37	24	45	16	15	46	31	30	29	31

[a]Odds ratio (CI) *P* value. The odds ratio with the corresponding 95% confidence interval (CI) for each variable was calculated by univariate analysis using Fisher's exact test. For zero values, the odds ratio was calculated by adding 0.5 to each value. ns: no significant association.
[b]High/Low determined with a cut-off equal to the mean halo diameter.
[c]Virulence level determined with a cut-off equal to the mean time (in days) to reach a mortality of 50% (LT50).

Table S5. Association of *rpf* types with virulence-related phenotypes.

rpf types	Number of isolates									
	Exoprotease production[b]		Twitching motility[b]		Biofilm		Virulence in *C. elegans*[c]		Virulence in *G. mellonella*[c]	
	High	Low	High	Low	Strong	Moderate-weak	Weak (LT50>7)	Moderate (LT50<7)	Moderate (LT50>1.2)	Strong (LT50<1.2)
rpf-1	27	9	23	13	3	33	20	16	19	16
rpf-2	10	15	18	7	12	13	11	14	10	15
Association[a]	4.5 (1.5-13.5) *P*=0.0082		ns		0.098 (0.02-0.41) *P*=0.0007		ns		ns	
Total	37	24	41	20	15	46	31	30	29	31

[a]Odds ratio (CI) *P* value. The odds ratio with the corresponding 95% confidence interval (CI) for each variable was calculated by univariate analysis using Fisher's exact test. For zero values, the odds ratio was calculated by adding 0.5 to each value. ns: no significant association. For associations with *rpf* typing, the odds ratio are for the *rpf*-1 variant.
[b]High/Low determined with a cut-off equal to the mean halo diameter.
[c]Virulence level determined with a cut-off equal to the mean time (in days) to reach a mortality of 50% (LT50).

Supplementary Figures

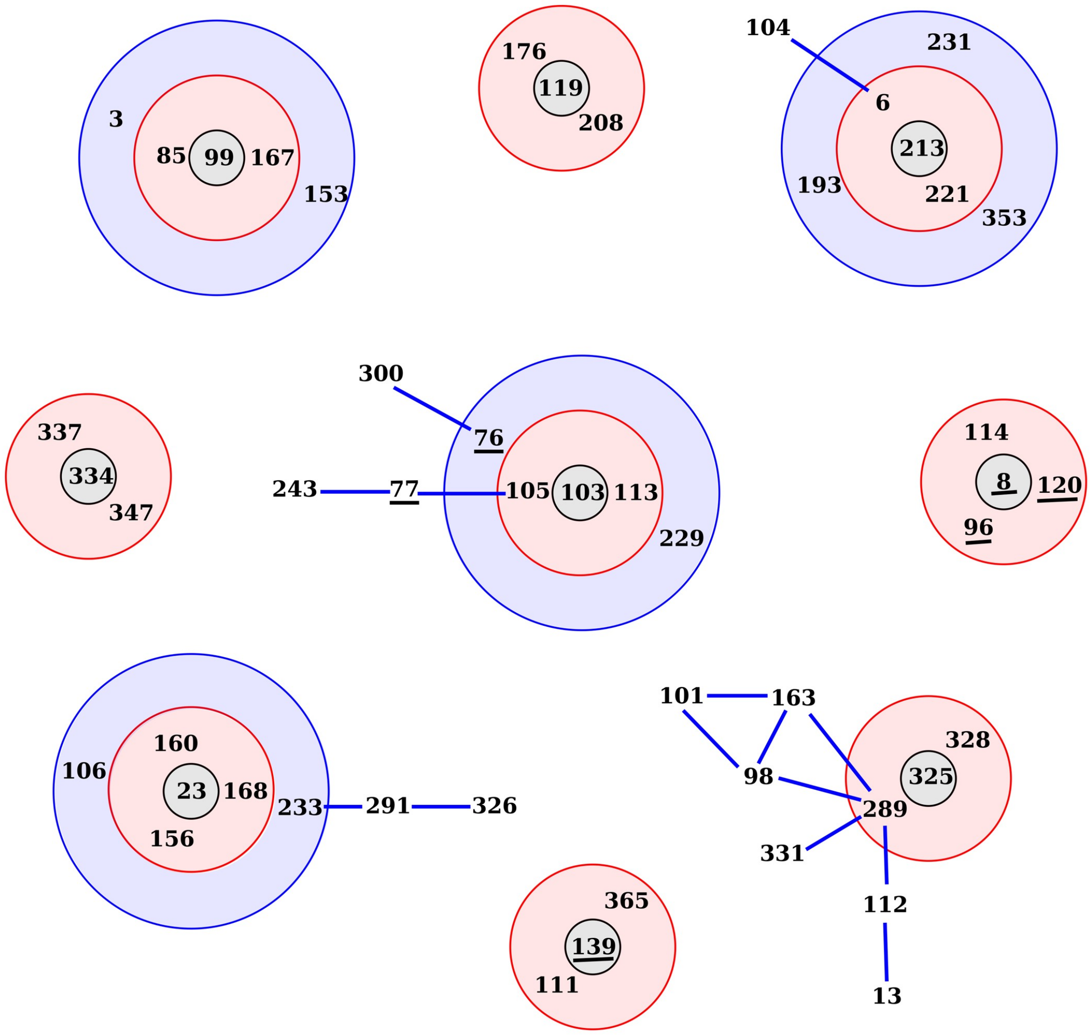

Figure S1. Population structure of *S. maltophilia*. Analysis of clonal groups using the BURST algorithm for 367 sequence types (STs) in the *S. maltophilia* MLST database at the time of analysis (Septembre, 2019). Only clonal groups where a founder (central ST) has been predicted are shown. Strains of the present study that form part of clonal groups belong to underlined STs. Gray circles (e.g. ST-8 and ST-139) represent predicted founder STs for a clonal group. Red circles identify single locus variants (STs sharing 6 identical alleles with the founder) and blue circles identify double locus variants (STs sharing 5 identical alleles with the founder). Outside the circles are satellite STs that vary by more than two loci from the central type, linked by blue lines to the STs with which they share 5 or 6 MLST loci.

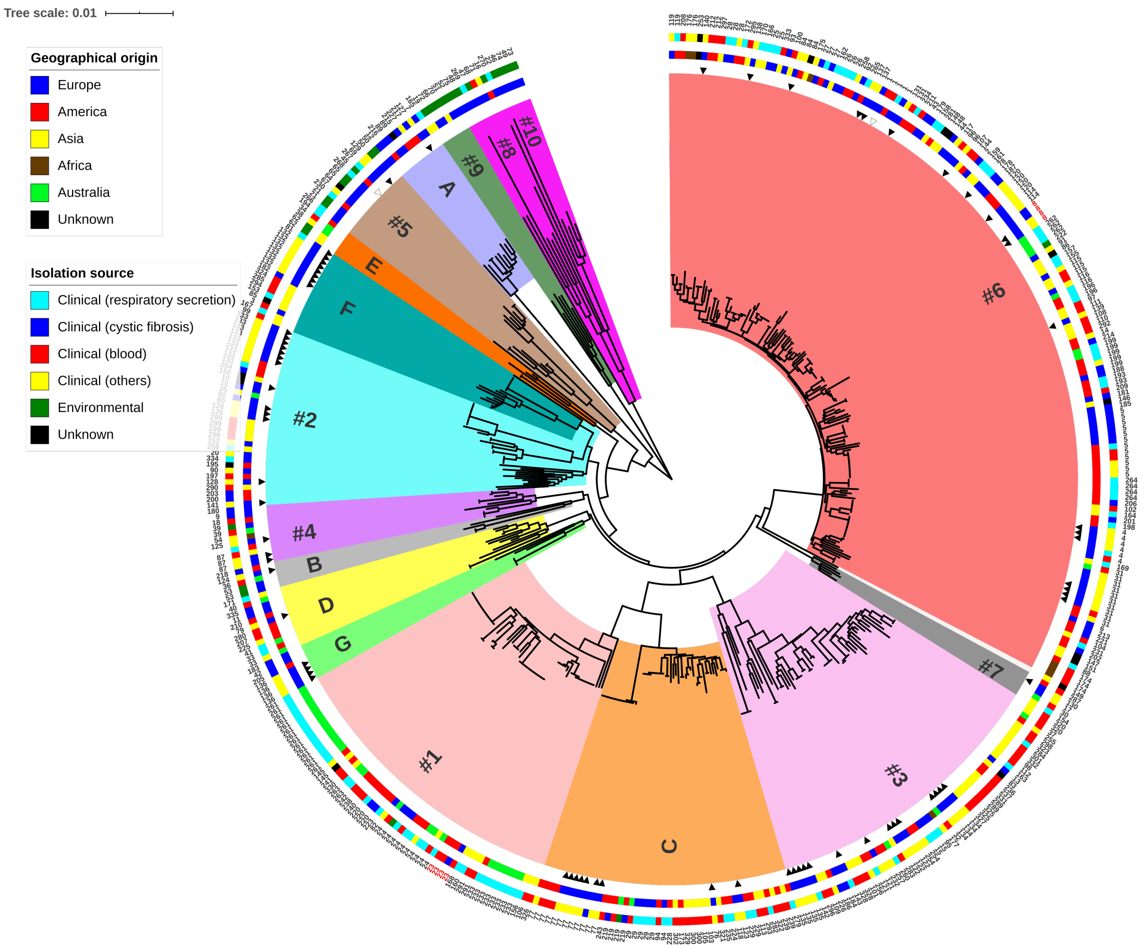

Figure S2. Global phylogenetic analysis of *S. maltophilia*. Neighbor-joining tree based on concatenated MLST gene sequences from 522 isolates in the MLST database at the time of analysis (September, 2019). The ST of each isolate is indicated with a number and displayed outside the tree. Branch colours indicate genomic groups according to Kaiser *et al*. (2009), Hauben *et al*. (1999) and Mercier-Darty *et al*. (2020). Coloured datasets outside the tree represent the region of origin (inner circle) and the isolation source (outer circle) with colours according to the legend on the left. Black arrowheads indicate the strains analyzed in this work. Reference strains K279a and R551-3 are indicated with empty arrowheads within genogroups #6 and #5 respectively.

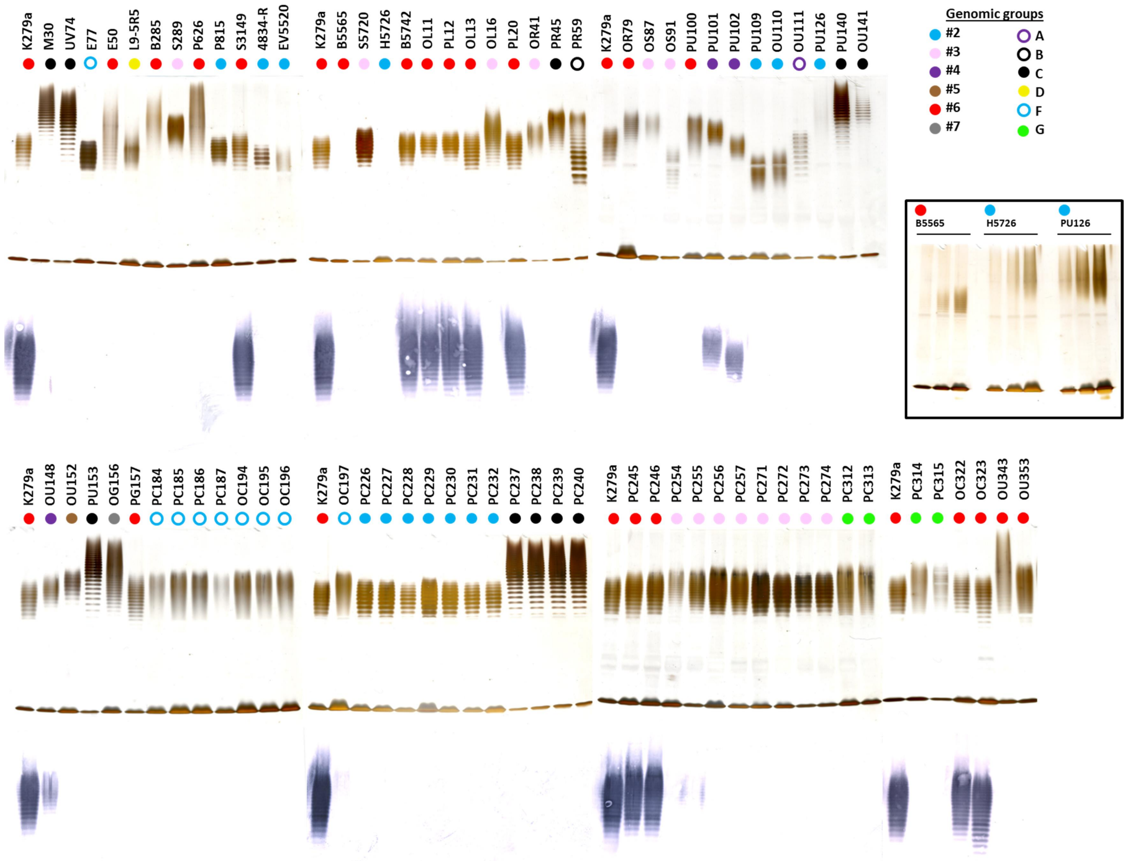

Figure S3. LPS profile typing and O-serotyping of *S. maltophilia* strains. LPS preparations from proteinase-K digested whole-cell lysates were separated on 12% SDS-PAGE gels and stained with silver nitrate (top panels). Corresponding immunoblots were developed with an antiserum specific for the O-polysaccharide of *S. maltophilia* K279a (bottom panels). The whole-cell lysate of K279a was used in each first lane as a control. The *inset* shows the characteristic profiles of smooth-type LPS of the strains B5565, H5726 and PU126 by increasing the amounts of LPS in SDS-PAGE, whereas no ladder-like LPS patterns were apparent using standard quantities of the samples. The genomic groups are indicated above each lane.

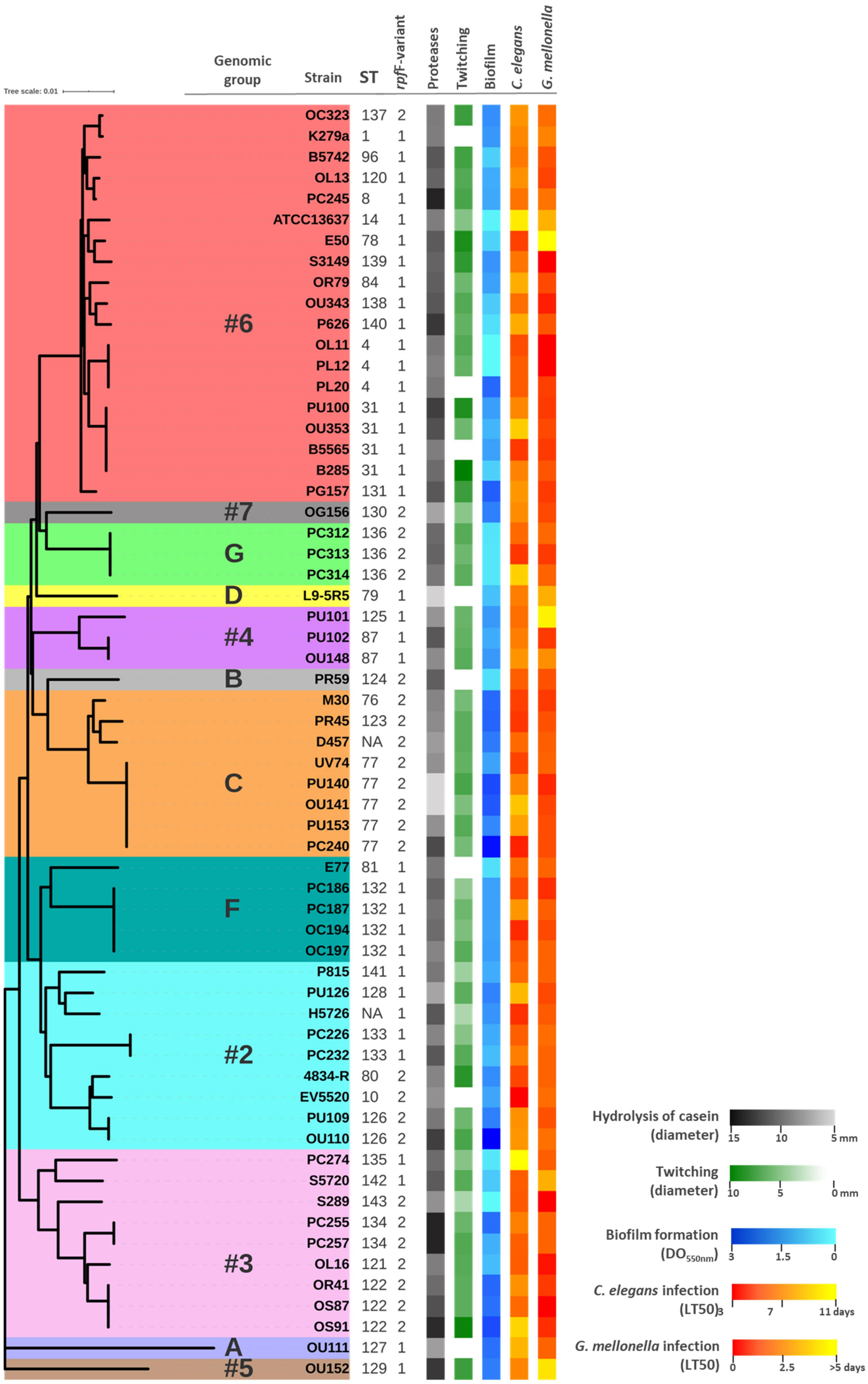

Figure S4. Correlation between genotypes and virulence-related phenotypes. Neighbour-joining tree based on the concatenated data for all seven MLST loci of the 61 “unique” S. maltophilia strains from this study. The ST of each isolate is indicated (NA means not assigned). Previously defined genomic groups are also indicated. Data for strain D457 was deduced from its whole genome sequence (NCBI Reference Sequence: NC_017671.1). There are five colored gradient datasets outside the tree for virulence-associated phenotypes. See legends on the side of the tree for each dataset's range of values.

Supplementary references

Gröschel, M., Meehan, C., Barilar, I., Diricks, M., Gonzaga, A., Steglich, M., et al. (2020). The phylogenetic landscape and nosocomial spread of the multidrug-resistant opportunist Stenotrophomonas maltophilia. *Nat. Commun.*, in press.

Hauben, L., Vauterin, L., Moore, E. R., Hoste, B., and Swings, J. (1999). Genomic diversity of the genus Stenotrophomonas. *Int. J. Syst. Bacteriol.* 49 Pt 4, 1749–1760. doi:10.1099/00207713-49-4-1749.

Huedo, P., Yero, D., Martínez-Servat, S., Estibariz, I., Planell, R., Martínez, P., et al. (2014). Two different rpf clusters distributed among a population of Stenotrophomonas maltophilia clinical strains display differential diffusible signal factor production and virulence regulation. *J. Bacteriol.* 196, 2431–2442. doi:10.1128/JB.01540-14.

Kaiser, S., Biehler, K., and Jonas, D. (2009). A Stenotrophomonas maltophilia multilocus sequence typing scheme for inferring population structure. *J. Bacteriol.* 191, 2934–2943. doi:10.1128/JB.00892-08.

Mercier-Darty, M., Royer, G., Lamy, B., Charron, C., Lemenand, O., Gomart, C., et al. (2020). Comparative whole genome phylogeny of animal, environmental, and human strains confirms the genogroup organization and diversity of the Stenotrophomonas maltophilia complex. *Appl. Environ. Microbiol.* doi:10.1128/AEM.02919-19.
